# Sustainable and high-level microbial production of plant hemoglobin in *Corynebacterium glutamicum*

**DOI:** 10.1186/s13068-023-02337-9

**Published:** 2023-05-11

**Authors:** Mengmeng Wang, Zhong Shi, Ning Gao, Yingyu Zhou, Xiaomeng Ni, Jiuzhou Chen, Jiao Liu, Wenjuan Zhou, Xuan Guo, Bo Xin, Yanbing Shen, Yu Wang, Ping Zheng, Jibin Sun

**Affiliations:** 1 College of Biotechnology, Tianjin University of Science and Technology, Tianjin, 300222 China; 2 Key Laboratory of Engineering Biology for Low-Carbon Manufacturing, Tianjin Institute of Industrial Biotechnology, Chinese Academy of Sciences, Tianjin, 300308 China; 3 National Technology Innovation Center of Synthetic Biology, Tianjin, 300308 China; 4 University of Chinese Academy of Sciences, Beijing, 100049 China

**Keywords:** Hemoglobin, Heme, Heterologous expression, *Corynebacterium glutamicum*, Meat analogs

## Abstract

**Background:**

Plant hemoglobin shows great potential as a food additive to circumvent the controversy of using animal materials. Microbial fermentation with engineered microorganisms is considered as a promising strategy for sustainable production of hemoglobin. As an endotoxin-free and GRAS (generally regarded as safe) bacterium, *Corynebacterium glutamicum* is an attractive host for hemoglobin biosynthesis.

**Results:**

Herein, *C. glutamicum* was engineered to efficiently produce plant hemoglobin. Hemoglobin genes from different sources including soybean and maize were selected and subjected to codon optimization*.* Interestingly, some candidates optimized for the codon usage bias of *Escherichia coli* outperformed those for *C. glutamicum* regarding the heterologous expression in *C. glutamicum*. Then, saturated synonymous mutation of the N-terminal coding sequences of hemoglobin genes and fluorescence-based high-throughput screening produced variants with 1.66- to 3.45-fold increase in hemoglobin expression level. To avoid the use of toxic inducers, such as isopropyl-β-d-thiogalactopyranoside, two native inducible expression systems based on food additives propionate and gluconate were developed. Promoter engineering improved the hemoglobin expression level by 2.2- to 12.2-fold. Combination of these strategies and plasmid copy number modification allowed intracellular production of hemoglobin up to approximately 20% of total protein. Transcriptome and proteome analyses of the hemoglobin-producing strain revealed the cellular response to excess hemoglobin accumulation. Several genes were identified as potential targets for further enhancing hemoglobin production.

**Conclusions:**

In this study, production of plant hemoglobin in *C. glutamicum* was systematically engineered by combining codon optimization, promoter engineering, plasmid copy number modification, and multi-omics-guided novel target discovery. This study offers useful design principles to genetically engineer *C. glutamicum* for the production of hemoglobin and other recombinant proteins.

**Supplementary Information:**

The online version contains supplementary material available at 10.1186/s13068-023-02337-9.

## Introduction

The Food and Agriculture Organization forecasts that the demand for meat will increase by 70% by 2050, which will be a severe problem considering the resource and environmental stress caused by the resource-intensive factory farming [1]. As an alternative, it is an irresistible trend for meat analogs playing an essential part of our daily diet [2]. Meat analogs are culinary products that mimic the appearance and flavor of meat and can be roughly divided into two categories: cell-based and plant-based meat analogs. Currently, imparting an acceptable flavor and texture to meat analogs for customers is among the biggest challenges for food manufacturers. Heme-containing proteins such as hemoglobin are already used in meat products as natural color enhancers, binders, or fat replacers [3]. The addition of heme-containing proteins in meat analogs also shows the potential to recreate natural meat taste and flavor [4]. In 2019, leghemoglobin (hemoglobin from *Glycine max*) produced by recombinant *Pichia pastoris* (also known as *Komagataella phaffii*) was authorized by the United States Food and Drug Administration as a color additive [5, 6]. Besides soybean, hemoglobin proteins widely exist in many plants, such as maize and rice, which are daily consumed in the human diet [7]. Therefore, plant hemoglobin produced by microbial fermentation shows great potential as a food additive to circumvent the controversy of using animal materials.

Only *Escherichia coli*, *P. pastoris*, and *Saccharomyces cerevisiae* have been engineered for sustainable production of hemoglobin from renewable resources. *E. coli* is considered the most convenient host for synthesizing hemoglobin, considering the effective genetic manipulation and low culture cost [6]. Most studies in *E. coli* focused on human hemoglobin which has potential application in transfusion therapy [8]. To overcome the shortage of internal heme, hemin or heme precursor 5-aminolevulinic (ALA) was exogenously supplemented. The heme transport system was usually enhanced to facilitate hemin uptake [9, 10]. Using these strategies, the highest titer of intracellular human hemoglobin (α2β2) in *E. coli* reached 6.56 g/L [11]. Despite the high production level, the application of hemoglobin produced by *E. coli* in food is limited due to the potential risk of endotoxin. Besides *E. coli*, GRAS (generally recognized as safe) yeast strains have also been selected for hemoglobin production. Impossible Foods Inc. explored and patented a commercial *P. pastoris* strain that efficiently produced leghemoglobin [12]. After ultrafiltration-based purification from cell lysate, the purity of hemoglobin reached over 65% and the safety was validated through a series of food allergy and toxicology tests [4]. Lately, *P. pastoris* was engineered for secretory expression of porcine myoglobin. By choosing a suitable host, signal peptide, and modified constitutive promoter, the titer of myoglobin reached 285.42 mg/L in fed-batch fermentations with feeding 150 mg/L of hemin [13]. Shao et al. engineered a *P. pastoris* strain capable of producing secretory leghemoglobin through gene dosage optimization and heme pathway consolidation. The highest leghemoglobin titer reached 3.5 g/L in high-density fermentation in a 10-L bioreactor [14]. In *S. cerevisiae*, through balancing globin expression and heme biosynthesis by overexpressing key biosynthetic genes, the expression level of human hemoglobin was improved to 4.09% of total intracellular proteins [15]. Further engineering of the heterologous protein degradation pathway resulted in the accumulation of human hemoglobin up to ∼18% of the total intracellular proteins [16]. Xue et al. systematically engineered *S. cerevisiae* for production of several hemoglobin and myoglobin proteins by optimizing the inducible expression strategy and enhancing native heme biosynthesis. Hemoglobin proteins of soybean and clover and myoglobin proteins of bovine and porcine were produced with titers up to 108.2 mg/L [17].

*Corynebacterium glutamicum* is an endotoxin-free and GRAS Gram-positive bacterium, which has been used for industrial production of various amino acids for over half a century [18, 19]. Recently, *C. glutamicum* has been recognized as an attractive host for secretory production of recombinant proteins due to the readily available genetic engineering tools [20–23], rapid growth in minimal media, and well-established industrial-scale fermentation technology [24, 25]. With discovery of signal peptides, metabolic engineering, and high cell density cultivation, the secretory production of some recombinant proteins reached gram per liter level [26, 27]. Besides, *C. glutamicum* has been engineered to synthesize high levels of free heme (242.95 mg/L) and its precursor ALA (18.5 g/L) [28, 29]. Therefore, *C. glutamicum* is an attractive host for hemoglobin biosynthesis.

In this study, we report high-level production of plant hemoglobin in *C. glutamicum*. Hemoglobin genes from different sources were selected for expression in *C. glutamicum*. Using a high-throughput screening method based on the hemoglobin–green fluorescent protein (GFP) fusion, the coding sequence of hemoglobin proteins, native inducible promoter, and plasmid copy number were systematically optimized for high-level hemoglobin expression. In addition, transcriptome and proteome analyses were employed to investigate the effects of hemoglobin overproduction on cellular metabolism and identify novel targets for future engineering.

## Results

### Selection of hemoglobin genes from different sources for expression in *C. glutamicum*

**Fig. 1:**
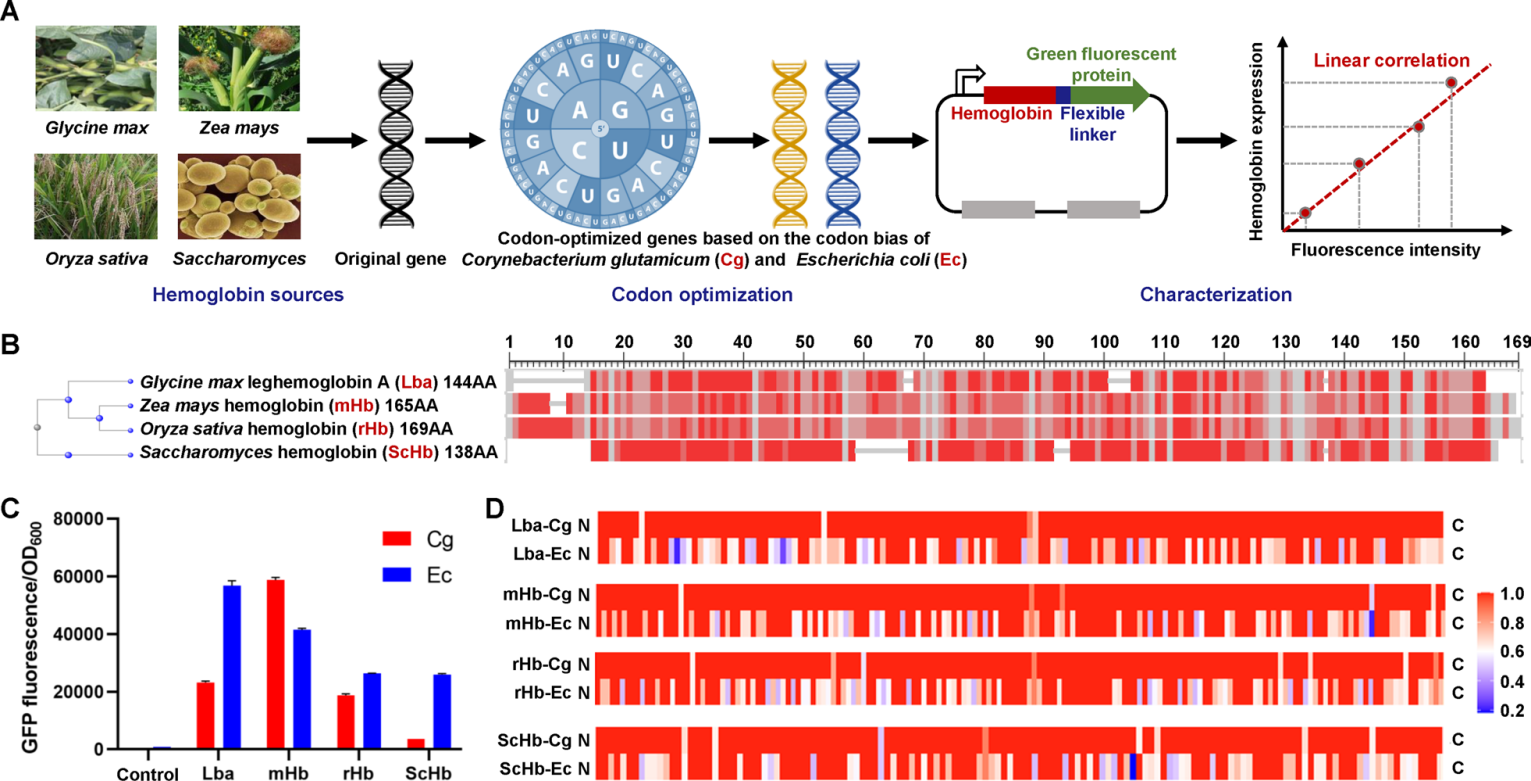
Hemoglobin genes from different sources and codon optimization. **A** Schematic diagram of gene selection, codon optimization, and characterization of hemoglobin expression in *C. glutamicum*. **B** Homology analysis of hemoglobin sequences from different sources. **C** Expression of hemoglobin genes from different sources after codon optimization. The hemoglobin expression levels were characterized by measuring the fluorescence intensities of hemoglobin–GFP fusion. Cg and Ec represent codon optimization according to the codon usage bias of *C. glutamicum* and *E. coli*, respectively. Values and error bars represent means and standard deviations (*n* = 3). **D** Relative codon adaptiveness of optimized genes with the codon usage frequency of *C. glutamicum* genome as a reference. For the codons encoding an amino acid, the relative codon adaptiveness of the codon with the highest usage frequency was set as 1. The relative codon adaptiveness of other codons for this amino acid was calculated based on their usage frequencies. For example, ATC, ATT, and ATA encode isoleucine and have usage frequencies of 34.2, 21.8, and 1.8 per thousand codons according to the Codon Usage Database (www.kazusa.or.jp/codon). The relative codon adaptiveness of ATC is set as 1, and those of ATT and ATA are calculated as 0.64 and 0.05, respectively

The leghemoglobin is a popular target for microbial production of recombinant hemoglobin [4, 14, 17]. Besides, the hemoglobins from maize (*Zea mays*), rice (*Oryza sativa*), and *S. cerevisiae* have also been characterized or heterologously expressed [30–32]. Considering that soybean, rice, and maize are common food crops and *S. cerevisiae* is widely used for baking and brewing, their hemoglobins are promising additives for meat analogs. Therefore, the encoding genes for the leghemoglobin A (Lba), maize hemoglobin (mHb), rice hemoglobin (rHb) and *S. cerevisiae* hemoglobin (ScHb) were selected for expression in *C. glutamicum* (Fig. 1A). Protein sequence alignment shows that the mHb and rHb are highly homologous and have a sequence identify of 72%. Lba has lower sequence identifies with mHb (40%) and rHb (44%). ScHb is independently evolved with the three plant hemoglobin proteins (Fig. 1B). The native genes of these four hemoglobin proteins were first synthesized based on their cDNA sequences and expressed in the wild-type *C. glutamicum* ATCC 13,032. For easy and accurate quantification of intracellular recombinant hemoglobin proteins, the synthesized genes were fused to a GFP gene with a flexible linker (GGGGS × 3) and inserted to the multiple cloning site of the plasmid pXMJ19 [33] under the control of IPTG-inducible promoter P_*tac*_ (Fig. 1A). IPTG was added to induce the expression of hemoglobin–GFP fusion and ALA and FeSO_4_ were supplemented to provide sufficient precursors for heme biosynthesis. With exogenous ALA and FeSO_4_, significantly elevated levels of extracellular heme were detected (Additional file 1: Fig. S1), suggesting that heme biosynthesis was enhanced and the heme efflux system was activated to maintain intracellular heme homeostasis [34]. Fluorescent intensity was measured to characterize hemoglobin expression. However, compared with the control strain harboring an empty plasmid, the strains overexpressing hemoglobin–GFP fusions produced very low-level GFP fluorescence intensities (Additional file 1: Fig. S2), suggesting the low expression level of the native hemoglobin genes.

Codon optimization is commonly necessary for expression of eukaryotic genes in prokaryotic hosts. However, it is suggested that the traditional codon optimization strategy based on codon usage bias (or frequency) shows uncertain effects in different hosts [35]. Therefore, we optimized the coding sequence of each hemoglobin protein according to the codon usage bias of *C. glutamicum* and *E. coli* (Fig. 1C and Additional file 1: Table S1). Taking Lba as an example, the Lba encoding genes with optimized codon usage of *C. glutamicum* and *E. coli* were designated as Lba-Cg and Lba-Ec, respectively. Expression tests were conducted using the wild-type *C. glutamicum* ATCC 13032 strain transformed with recombinant plasmids*.* Codon optimization overall increased the expression level of hemoglobin proteins. Interestingly, for Lba, rHb, and ScHb, the encoding genes optimized for *E. coli* outperformed those for *C. glutamicum* regarding the expression level in *C. glutamicum* (Fig. 1C). Lba-Ec showed a high fluorescence intensity of 56,701, which was 2.4-fold of Lba-Cg. rHb-Ec and ScHb-Ec had similar fluorescence intensities, which were approximately half of Lba-Ec but 1.4- and 7.3-fold higher than rHb-Cg and ScHb-Cg, respectively. Only for mHb, the *C. glutamicum* version showed higher expression level than the *E. coli* version. mHb-Cg had a similar fluorescence intensity (58,748) with Lba-Ec, which was 1.4-fold higher than mHb-Ec (Fig. 1C). The sequences of all the genes were analyzed with the codon usage frequency of *C. glutamicum* genome as a reference. The *C. glutamicum* versions basically have higher codon usage frequencies than the *E. coli* versions (Fig. 1D). However, such high codon usage frequencies were not always successfully transferred into high protein expression levels. Lba-Ec and mHb-Cg had the highest expression level and thus were chosen as candidates for hemoglobin expression in *C. glutamicum*.

### Optimization of N-terminal coding sequence by saturated synonymous mutation

**Fig. 2:**
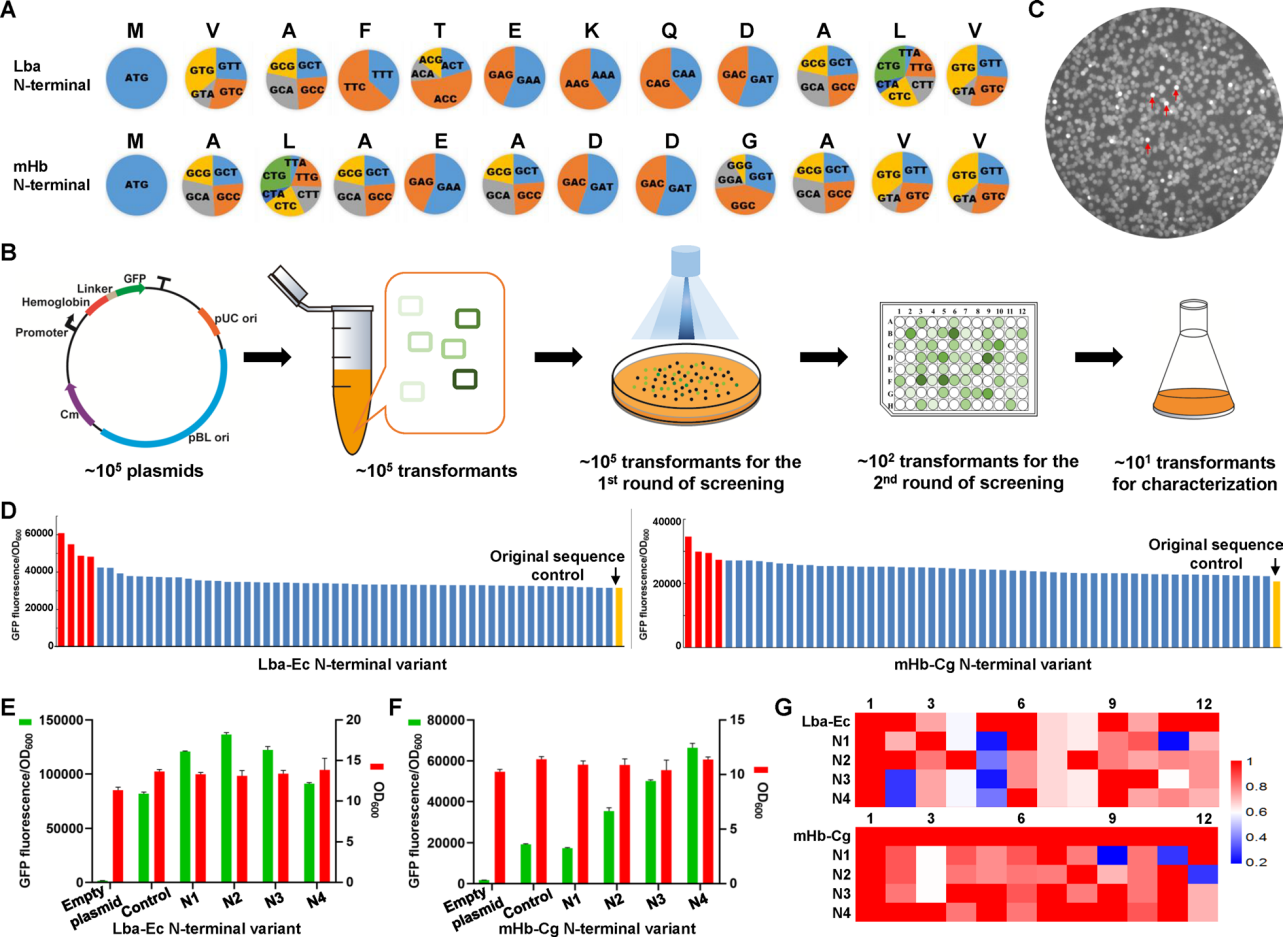
Optimization of the N-terminal coding sequence of hemoglobin. **A** Schematic diagram of saturated synonymous mutation for the first 12 codons of Lba-Ec and mHb-Cg. The pie charts represent the codon usage frequency in *C. glutamicum*. **B** Schematic diagram of NCS library construction and screening using the fluorescence imaging system. **C** Representative image of the screening agar plates got by the fluorescence imaging system. Colonies with relatively high fluorescence intensities are marked with red arrows. **D** Second round of screening by cultivation in 96-well plates. **E** Characterization of the selected NCS variants from the Lba-Ec library by cultivation in shaking flasks. To remove the possible mutations in the plasmid backbone or the chromosome that were randomly introduced during library construction and screening, the expressing plasmids were reconstructed based on the sequencing data and transformed into *C. glutamicum* for test. **F** Characterization of the selected NCS variants from the mHb-Cg library by cultivation in shaking flasks. To remove the possible mutations in the plasmid backbone or the chromosome that were randomly introduced during library construction and screening, the expressing plasmids were reconstructed based on the sequencing data and transformed into *C. glutamicum* for test. **G** Codon relative adaptiveness of the first 12 codons of the selected variants with the codon usage frequency of *C. glutamicum* genome as a reference. The hemoglobin expression levels were characterized by measuring the fluorescence intensities of hemoglobin–GFP fusion. Values and error bars represent means and standard deviations (*n* = 3)

**Fig. 3:**
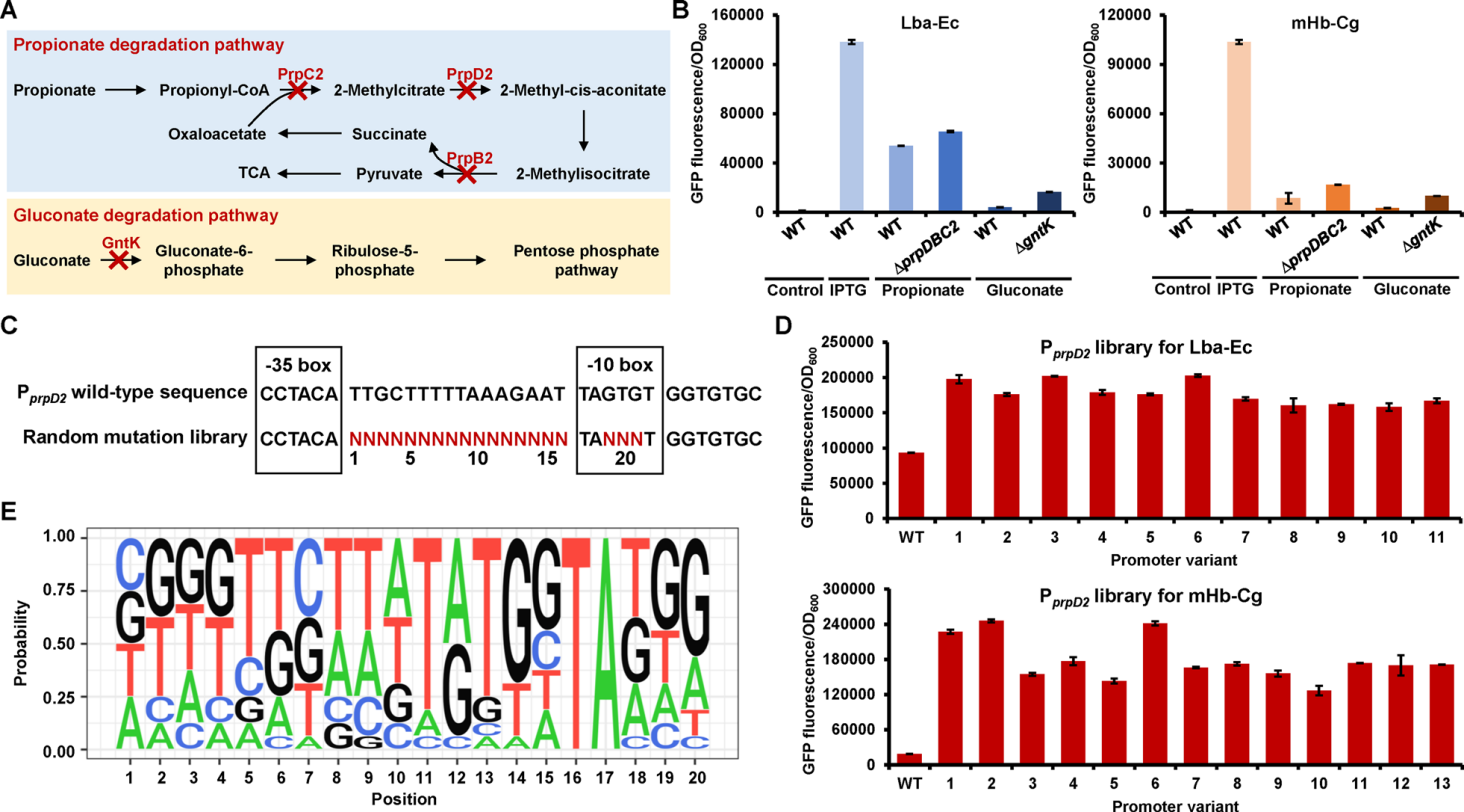
Engineering of native promoters for inducible hemoglobin expression. **A** Catabolic pathway of propionate and gluconate in *C. glutamicum*. The red crosses represent gene knock-out to block the degradation of propionate and gluconate. **B** Comparison of different inducible promoters for hemoglobin expression. WT, the wild-type *C. glutamicum* strain. ∆*prpDBC2*, the *C. glutamicum* mutant with propionate catabolic genes *prpDBC2* deleted. ∆*gntK*, the *C. glutamicum* mutant with gluconate catabolic gene *gntK* deleted. Control represents the wild-type strain transformed with an empty plasmid. The pXMJ19-Lba-Ec-*gfp* and the pXMJ19-mHb-Cg-*gfp* plasmids for IPTG-inducible expression of the hemoglobin–GFP fusions were transformed into the wild-type strain. The pXMJ19-P_*prpD2*_-Lba-Ec-*gfp* and the pXMJ19-P_*prpD2*_-mHb-Cg-*gfp* plasmids for propionate-inducible expression of the hemoglobin–GFP fusions were transformed into the wild-type strain and the ∆*prpDBC2* strain. The pXMJ19-P_*gntK*_-Lba-Ec-*gfp* and the pXMJ19-P_*gntK*_-mHb-Cg-*gfp* plasmids for gluconate-inducible expression of the hemoglobin–GFP fusions were transformed into the wild-type strain and the ∆*gntK* strain. **C** Schematic diagram of the designed P_*prpD2*_ promoter libraries. The introduced degenerate codons are highlighted in red. **D** Characterization of selected P_*prpD2*_ promoter variants for hemoglobin expression. WT represents the wild-type P_*prpD2*_ promoter and the rest are promoter variants selected from the promoter libraries. To remove the possible mutations in the plasmid backbone or the chromosome that were randomly introduced during library construction and screening, the expressing plasmids were reconstructed based on the sequencing data and transformed into *C. glutamicum* for test. **E** Analysis of the sequence characteristics of the strong P_*prpD2*_ promoter variants selected from the random mutation library. The hemoglobin expression levels were characterized by measuring the fluorescence intensities of hemoglobin–GFP fusion. Values and error bars represent means and standard deviations (*n* = 3)

Codon usage of the N-terminal coding sequence (NCS) was reported to greatly affect the stability of gene transcript and the efficiency of translational initiation, which determined the protein expression level [36]. However, there is a lack of a reliable principle to guide the rational design of NCS. Therefore, to optimize the NCS for improving hemoglobin expression, we replaced the first 12 codons of Lba-Ec and mHb-Cg with their synonymous codons using primers containing degenerate nucleotides (Fig. 2A). An NCS library with saturated synonymous mutations was constructed for Lba-Ec and mHb-Cg, respectively. The hemoglobin–GFP fusion allowed high-throughput screening of NCS variants with enhanced expression using a fluorescence imaging system (Fig. 2B). Variants with high fluorescence intensities observed in the agar plates were cultured in 96-well plates for a second round of screening (Fig. 2C). The best four variants with the highest fluorescence intensities were characterized in shaking flask cultivation (Fig. 2D). Selected Lba-Ec variants showed 1.10- to 1.66-fold improvement (Fig. 2E) and mHb-Cg variants showed 1.85- to 3.45-fold improvement (Fig. 2F). Sequencing of the selected NCS variants suggests no significant correlation between the expression level and the codon usage frequency in *C. glutamicum* (Fig. 3G and Additional file 1: Table S2)*.* However, it can be observed that several codons with high usage frequencies are replaced with codon with relatively low usage frequency or even rare codons.

### Characterization and engineering of native promoters for high-level inducible expression

Since the produced hemoglobin is to be used as a food additive, the use of toxic inducer IPTG is not allowed. To avoid the use of IPTG and the introduction of heterologous genetic elements, such as the LacI/P_*tac*_ system, we selected two endogenous inducible expression system with commonly used food additives as inducers for hemoglobin expression in *C. glutamicum*. The PrpR/P_*prpD2*_ system originates from the propionate metabolism and is induced by propionate. PrpR is the transcriptional activator of the gene cluster *prpDBC2* that encoding key enzymes catalyzing propionate to pyruvate for metabolism (Fig. 3A). The promoter/operator region of the *prpDBC2* gene cluster has been identified, allowing the development of propionate-inducible expression system [37]. The GntR/P_*gntK*_ system originates from the gluconate metabolism and is induced by gluconate [38]. GntR-type transcriptional regulators, GntR1 and GntR2, strongly repress the gluconate transport and metabolic genes including the gluconate permease (*gntP*), gluconate kinase (*gntK*), and 6-phosphogluconate dehydrogenase (*gnd*). Gluconate interferes with binding of GntR1 and GntR2 to their target promoters, such as P_*gntK*_, leading to a derepression of target genes [39].

Although these two systems allowed hemoglobin Lba-Ec and mHb-Cg expression in *C. glutamicum*, the expression level was significantly lower than that obtained using the IPTG-inducible P_*tac*_ promoter (Fig. 3B). Propionate can be metabolized to pyruvate and enter the tricarboxylic acid cycle [37], and gluconate can be utilized as a carbon source via the oxidative pentose phosphate pathway [38] (Fig. 3A). Degradation of the inducers may lower the induction strength and negatively affect protein expression. To reduce the degradation of inducers, the *prpDBC* gene cluster and *gntK* gene were deleted, resulting in mutants ∆*prpDBC2* and ∆*gntK*, respectively. Blocking the degradation of propionate and gluconate increased the expression of hemoglobin, whereas the expression levels were still lower than those obtained by the IPTG-inducible system (Fig. 3B). Overall, the induction strength of propionate inducible P_*prpD2*_ was higher than that of gluconate inducible P_*gntK*_ (Fig. 3B), so P_*prpD2*_ was selected for subsequent engineering.

To increase the strength of the P_*prpD2*_ promoter, we constructed two random mutation libraries for Lba-Ec and mHb-Cg expression by mutating the spacer region between the -10 and -35 boxes and part of the -10 box of the P_*prpD2*_ promoter. The libraries were screened using the fluorescence imaging system and cultivation in 96-well plates. Selected variants were characterized by cultivation in shake flasks. From the library for Lba-Ec expression, variants with 1.7- to 2.2-fold higher expression levels than the wild-type P_*prpD2*_ promoter were obtained. From the library for mHb-Cg expression, variants with 6.7- to 12.8-fold improvement were obtained (Fig. 3D and Additional file 1: Table S3). Using the best P_*prpD2*_ variants for Lba-Ec and mHb-Cg expression, their expression levels were 1.46-fold and 2.37-fold higher than those obtained using the IPTG-inducible P_*tac*_ promoter, respectively. Sequence analysis of the screened promoter variants with increased strength suggests good sequence diversity. The enrichment of T at the positions 11 and 13 and the enrichment of G at position 14 were observed (Fig. 3E).

### Combinatorial optimization to further enhance hemoglobin expression

**Fig. 4:**
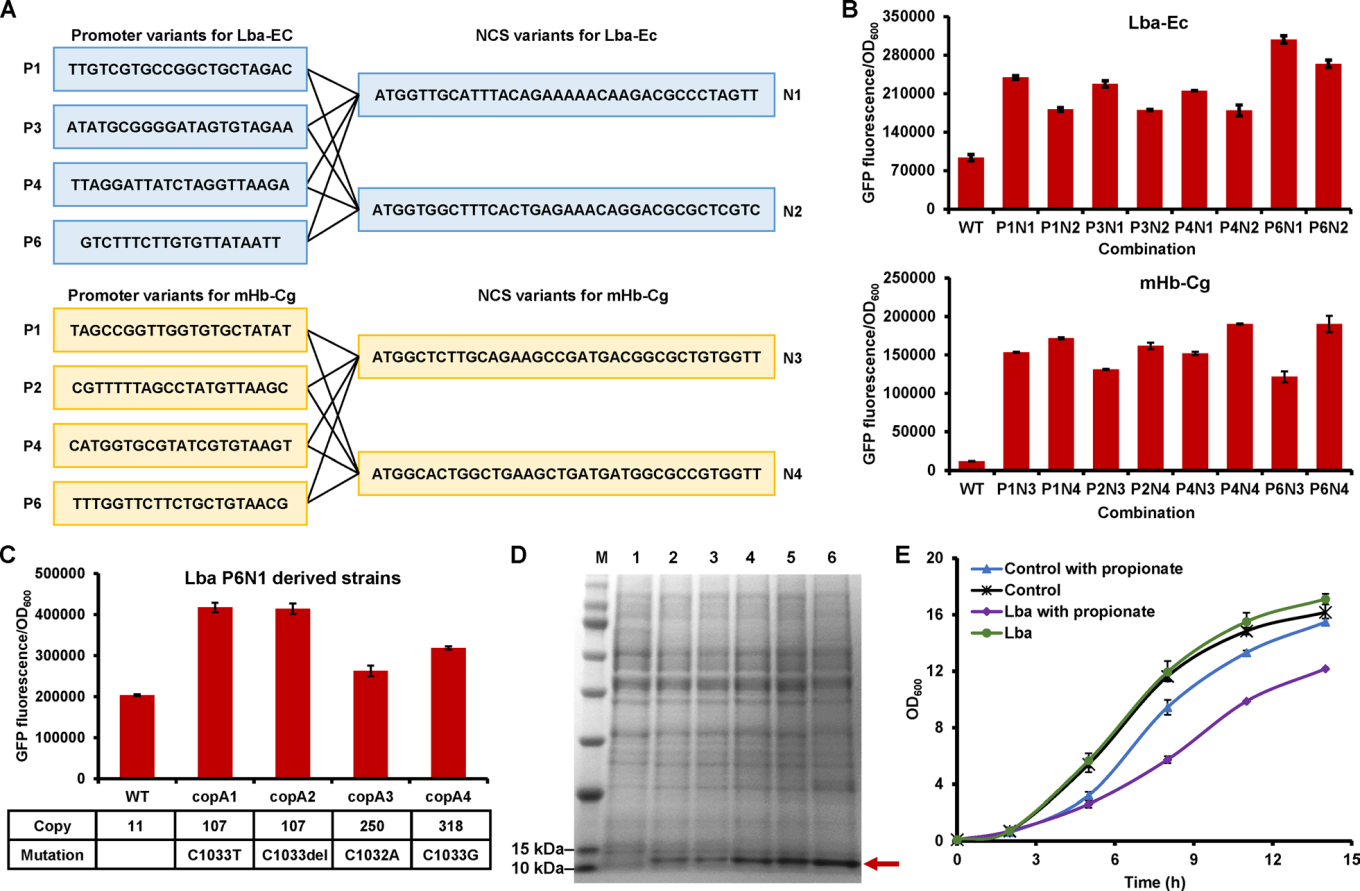
Combinatorial optimization of multiple elements for enhancing hemoglobin expression. **A** Schematic diagram of combination of P_*prpD2*_ promoter variants with hemoglobin NCS variants. **B** Effects of combination of P_*prpD2*_ promoter variants with hemoglobin NCS variants on hemoglobin expression. **C** Effects of plasmid copy number modification on hemoglobin expression. **D** Analysis of intracellular hemoglobin expression by SDS–PAGE. M, protein marker. 1, empty plasmid control. 2, Lba-Ec with unoptimized NCS and the wild-type P_*prpD2*_ promoter. 3, Lba-Ec with the optimized NCS variant (N1) and the wild-type P_*prpD2*_ promoter. 4, Lba-Ec with the unoptimized NCS and the optimized P_*prpD2*_ promoter variant (P6). 5, Lba-Ec with the optimized NCS variant (N1) and the optimized P_*prpD2*_ promoter variant (P6). 6, Lba-Ec with the optimized NCS variant (N1) and the optimized P_*prpD2*_ promoter variant (P6) in a high-copy plasmid (*copA1* mutation, 107 copies). **E** Effects of hemoglobin expression on cell growth. The *C. glutamicum* strain with an empty plasmid was used as the control. The hemoglobin expression levels were characterized by measuring the fluorescence intensities of hemoglobin–GFP fusion. Values and error bars represent means and standard deviations (*n* = 3)

The NCS of hemoglobin genes and the promoter for hemoglobin expression were individually optimized. To investigate whether the screened NCS and promoter variants have combinatorial effects on hemoglobin expression, combinations of these elements were constructed and tested (Fig. 4A). For Lba-Ec, the combinations showed 1.9- to 3.3-fold higher hemoglobin expression than the original Lba-Ec gene controlled by the wild-type P_*prpD2*_ promoter. For mHb-Cg, the combination showed 10.2- to 16.0-fold higher hemoglobin expression than the original mHb-Cg controlled by the wild-type P_*prpD2*_ promoter. According to the fluorescence intensities of the hemoglobin–GFP expressed using all these combinations, the Lba-P6N1 (Lba-Ec with the P_*prpD2*_ promoter variant P6 combined with the NCS variant N1) has the highest expression level (Fig. 4B).

The pXMJ19 plasmid used for hemoglobin expression only has a copy number of 11 in *C. glutamicum* [40]. We assume that increasing the copy number of pXMJ19 may be beneficial for expression of heterologous proteins. A previous study reported several mutations (base substitution or deletion) in the initiator protein encoding gene *copA* largely increased the plasmid copy number [40]. Four pXMJ19 variants with increased copy numbers ranging from 107 to 318 were constructed and used for expressing the Lba-Ec with optimized NCS and promoter. When the copy number was increased to 107, the highest expression level was about twice that of the original plasmid (Fig. 4C). However, when the plasmid copy number was increased to higher values, decreased expression levels of hemoglobin were observed. All the optimizations were evaluated using measuring the fluorescence intensity of the hemoglobin–GFP fusion. After obtaining the strain with the highest expression level of the hemoglobin–GFP fusion, we removed the GFP and constructed a series of plasmids for expressing Lba-Ec. Then, SDS–PAGE was used to analyze the expression level of Lba-Ec. The result shows that with the optimization of NCS, promoter, and plasmid copy number, the expression of soluble hemoglobin was gradually increased. According to the grayscale analysis using the software Image J and Gel-Pro Analyzer, the highest expression of hemoglobin was estimated to account for approximately 20% of the total protein (Fig. 4D). Besides, there is a good linear relation between the hemoglobin expression levels analyzed by SDS–PAGE and those analyzed by quantifying the fluorescence intensities of the hemoglobin–GFP fusion (Additional file 1: Fig. S3). The result suggests the reliability of quantifying the hemoglobin expression level by fusing the target protein to a fluorescent protein and measuring the fluorescence intensity. Using a heme assay kit, the heme-binding ratio of the produced hemoglobin was estimated to be 28%, which was comparable to the hemoglobins and myoglobins produced by *S. cerevisiae* (12–27%) [17].

### Multi-omics analyses for deciphering the effects of hemoglobin overproduction on host cells

Such a higher expression of intracellular proteins may have negative effects on cell growth. We then tested the cell growth of strains with or without overproduction of hemoglobin. Without propionate induction, the strain harboring hemoglobin expressing plasmid showed similar cell growth with the control strain harboring an empty plasmid. When propionate was added, a slight inhibition on cell growth of the control strain harboring an empty plasmid was observed (Fig. 4E). Since the *prpDBC2* cluster was deleted, propionate could be converted to propionyl-CoA but could not be further metabolized (Fig. 3A). One possible explanation to the growth retardation is that the CoA regeneration from propionyl-CoA might be affected. However, when hemoglobin expression was induced by propionate addition, serious inhibition on the cell growth was observed (Fig. 4E), suggesting that the overproduction of hemoglobin brought significant effects on cellular metabolism.

**Fig. 5:**
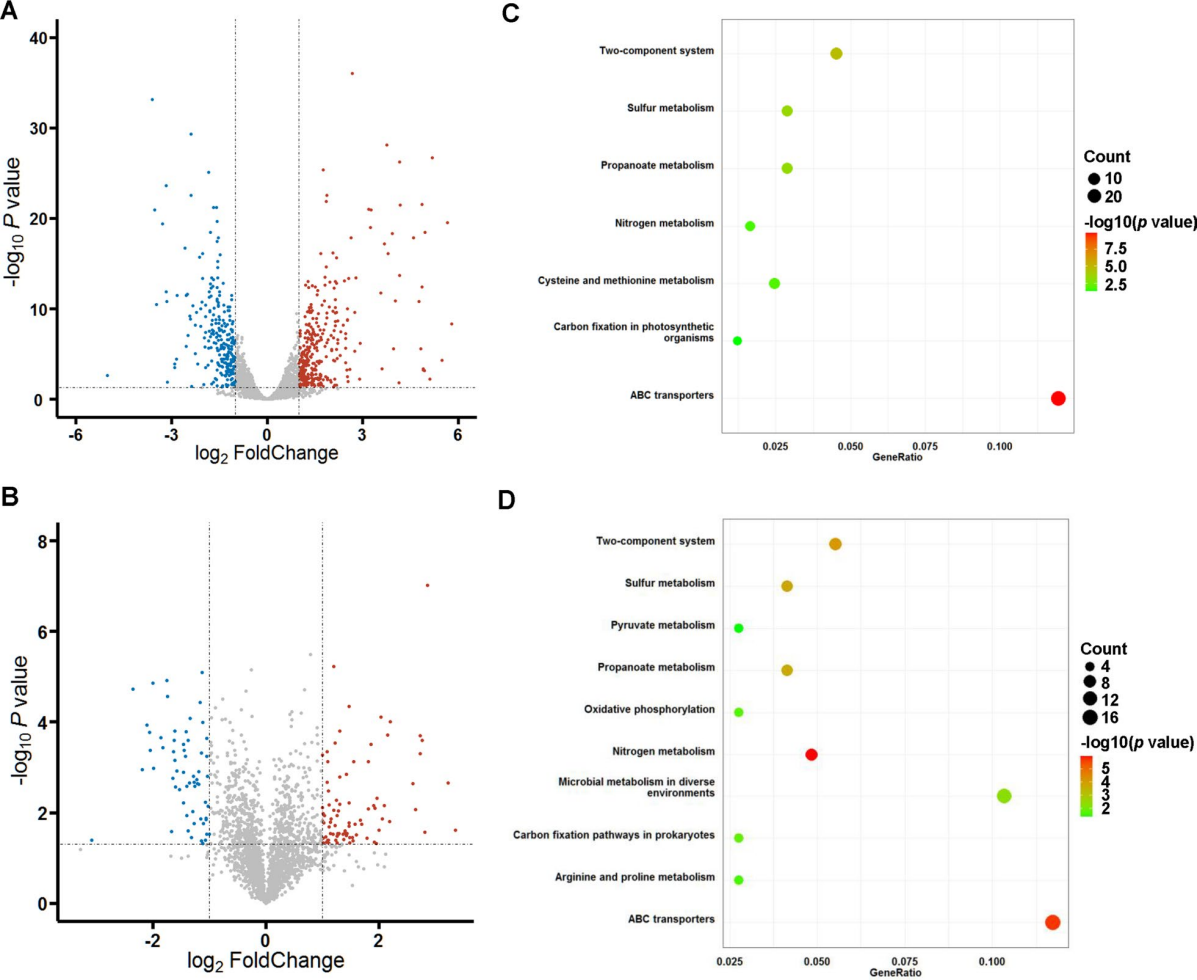
Transcriptomic and proteomic analyses of hemoglobin-overproducing *C. glutamicum* with the strain harboring an empty plasmid as a control. **A** Differentially expressed genes identified by the transcriptomic analysis. The red and blue circles represent up-regulated and down-regulated genes (log_2_(Fold change) > 1 or < − 1, *p* < 0.05 by two-tailed Student’s *t* test, *n* = 3), respectively.** B** Differentially expressed genes identified by the proteomic analysis. The red and blue circles represent up-regulated and down-regulated proteins (log_2_(Fold change) > 1 or < − 1, *p* < 0.05 by two-tailed Student’s *t* test, *n* = 3), respectively.** C** KEGG enrichment analysis of the differentially expressed genes in transcriptomic analysis. **D** KEGG enrichment analysis of the differentially expressed genes in proteomic analysis

To decipher the effects of hemoglobin expression on the host cells, the transcriptome and proteome of the strain overproducing hemoglobin were profiled with the strain harboring an empty plasmid as a control. RNA-seq identified 559 genes with at least twofold changes in transcription levels, including 316 up-regulated genes and 243 down-regulated genes (Fig. 5A and Additional file 2: Table S4). Proteomic analysis revealed 136 proteins with at least twofold changes in protein levels, including 59 up-regulated proteins and 77 down-regulated proteins (Fig. 5B and Additional file 2: Table S4). The differentially expressed genes were then classified using Kyoto Encyclopedia of Genes and Genomes (KEGG) annotation. In the KEGG enrichment analysis shows that two-component system, sulfur metabolism, propanoate metabolism, nitrogen metabolism, carbon fixation pathways, and ABC transporters were significantly enriched both in the transcriptomic and proteomic analyses (Fig. 5C, D).

### Multi-omics-guided screening of novel targets affecting hemoglobin expression

**Fig. 6:**
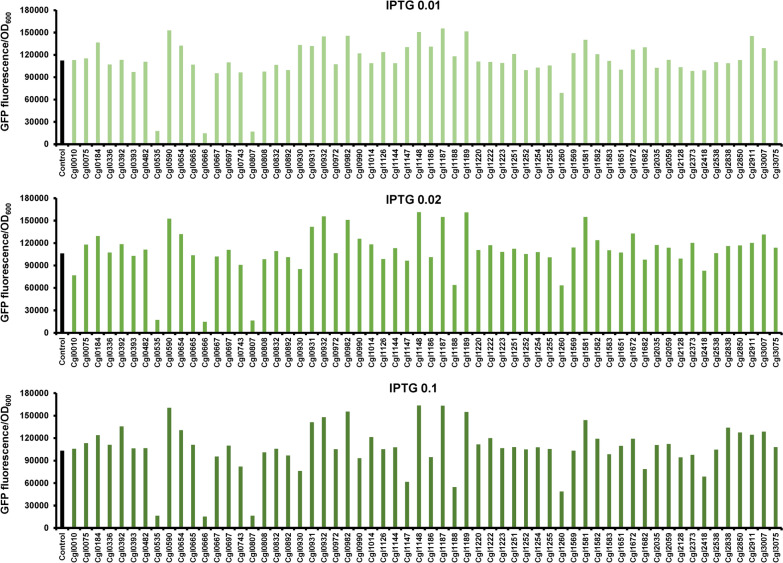
Effects of overexpression of selected genes on hemoglobin expression. Genes that were identified as differentially expressed genes both in the transcriptomic and proteomic analyses were overexpressed in plasmid pEC-XK99E and the effects on hemoglobin expression were determined. An empty pEC-XK99E plasmid was used as the control. Different IPTG concentrations (0.01, 0.02, and 0.1 mM) were used to induce the expression of selected genes. Propionate was added to induce hemoglobin Lba-Ec expression. The hemoglobin expression levels were characterized by measuring the fluorescence intensities of hemoglobin–GFP fusion

Although a large number of differentially expressed genes were identified by the transcriptomic and proteomic analyses, few of these genes are directly involved in the processes of protein biosynthesis or degradation. Thus, it is difficult to speculate the function of these genes in regulating heterologous production expression or responding to the stress caused by hemoglobin overproduction. We selected 60 differentially expressed genes, the transcriptional and protein levels of which were both changed by over twofold (log_2_(Fold change) > 1 or < − 1, *p* < 0.05), for further investigation. Gene overexpression plasmids for these selected genes were collected from an open reading frame library of *C. glutamicum* based on the plasmid pEC-XK99E [41] (laboratory stock, unpublished resources) and transformed into the hemoglobin Lba-Ec expressing strain*.* The selected genes are under the control of IPTG-inducible P_*trc*_ promoter and their expression levels can be adjusted by adding different concentrations of IPTG. Three IPTG concentrations (0.01, 0.02, and 0.1 mM) were used in the present study and different effects on hemoglobin expression were observed. A preliminary screening showed that overexpression of several genes improved the production of hemoglobin, while severe inhibition on hemoglobin expression was observed for a few other genes (Fig. 6). Finally, we identified several novel targets for improving hemoglobin production in *C. glutamicum,* including *cgl0590*, *cgl0930*, *cgl0931*, *cgl0932*, *cgl0982*, *cgl1148*, *cgl1187*, *cgl1189*, and *cgl1581*. Among these genes, half of them were predicted as membrane-bound transporter encoding genes (*cgl0930*, *cgl0931*, *cgl0932*, and *cgl1148* encoding ABC-type transporters and *cgl0590* encoding a putative permease). *cgl1187*, *cgl1189*, and *cgl1581* are involved in nitrogen metabolism and *cgl0982* is annotated as transcriptional regulators (Additional file 2: Table S4). According to the transcriptomic and proteomic analyses of the strain overproducing hemoglobin, most of these identified genes (*cgl0590*, *cgl0930*, *cgl0931*, *cgl0932*, *cgl1148*, *cgl1187*, *cgl1189*, and *cgl1581*) showed down-regulated expression levels (Additional file 2: Table S4). This result suggests that for certain reasons, high-level expression of hemoglobin repressed these genes and restoring or enhancing their expression could improve hemoglobin production.

## Discussion

Plant hemoglobin shows great potential as a food additive that imparts meaty flavor and color to meat analogs. In this study, we selected an endotoxin-free and GRAS bacterium *C. glutamicum* as a microbial cell factory for synthesis of plant hemoglobin. For food safety consideration, besides a GRAS microbial host, an inducible high-level expression system without the use of toxic inducer is also needed. Compared with *E. coli*, the inducible expression systems for *C. glutamicum*, especially those enabling high-level gene expression are limited. The LacI/P_*tac*_ system induced by the toxic compound IPTG is still the most popular choice for heterologous expression in *C. glutamicum*. This study provides two endogenous inducible expression systems with engineered promoters, the propionate-inducible PrpR/P_*prpD2*_ and the gluconate-inducible GntR/P_*gntK*_ systems, which use common food additives as inducers and enable even higher protein expression levels than the canonical IPTG-inducible LacI/P_*tac*_ system. Combined with optimization of gene codon, NCS, and plasmid copy number, production of hemoglobin was systematically optimized at the translational and transcriptional levels. Finally, the highest expression level of hemoglobin was estimated to account for approximately 20% of the total protein in *C. glutamicum.*

Although synonymous mutations were previously thought to be silent, a large body of evidence has demonstrated that codon usage can play major roles in determining gene expression levels and protein structures. Codon usage influences translation elongation speed and regulates translation efficiency and accuracy [42]. Therefore, the codon optimization strategy has been widely used in heterologous expression. However, the codon optimization for the plant hemoglobin genes based on the codon usage frequency of *C. glutamicum* did not lead to high-level protein expression in some of our cases. Unexpectedly, codon optimization according to the codon usage frequency of *E. coli* produced even higher expression levels in three out of four tests. The result suggests that the hemoglobin expression level has no significant correlation with the codon usage frequency in *C. glutamicum.* In addition to the codon usage frequency, codon optimization strategies based on other parameters, such as the tRNA copy number and tRNA abundance, have been developed. However, most tests of these new strategies are conducted in *E. coli* [43, 44], and little information is available for *C. glutamicum*. The NCS of a gene is considered as a determinant of mRNA secondary structure, stability, and translation initiation efficiency [42]. Considering the short length of NCS (about 10 codons), it is possible to construct a NCS library containing saturated synonymous mutations. The hemoglobin–GFP fusion allows high-throughput screening of desired variants with increased expression levels. Analysis of the selected NCSs suggested the introduction of a few codons with relatively low codon usage frequencies or even rare codons. The optimized NCS may promote the protein chain elongation by reducing ribosome collision and stacking [36].

The overproduction of heterologous protein may exert pressure on cell metabolism and hinder the expression of target protein in turn. The large number of differentially expressed genes identified by the transcriptomic and proteomic analyses support this hypothesis. Interestingly, the differentially expressed genes are closely related to two-component system, sulfur metabolism, nitrogen metabolism, carbon fixation pathways, and ABC transporters, which are not directly involved in heterologous protein expression. It seems that hemoglobin expression affects the transcriptional regulatory process, energy metabolism, and substance transport. Multi-omics-guided gene overexpression screening provided several gene targets affecting heterologous protein expression. Among these genes, *cgl0930*, *cgl0931*, and *cgl0932* locate in an operon (*cgl0930*–*cgl0934*, also known as *urtABCDE*) encoding the *C. glutamicum* urea uptake system [45]. Overexpression of *cgl0930*, *cgl0931*, *cgl0932*, and certain genes involved in nitrogen metabolism (such as *cgl1187*, *cgl1189*, and *cgl1581*) benefited hemoglobin production, suggesting the importance of the transport and metabolism of nitrogen source in protein overproduction. Recently, CRISPRi-microfluidics screening identified gene targets that can be engineered to enhance recombinant protein secretion in *C. glutamicum*, part of which are previously unknown targets involved in transmembrane transport, amino acid metabolism, and redox regulation [46]. These findings provide potential targets for further enhancing the production of hemoglobin and other recombinant proteins in *C. glutamicum*.

## Conclusions

In this study, we explored the potential of *C. glutamicum* for microbial production of plant hemoglobin. By combining multiple strategies, the product yield of intracellular hemoglobin in *C. glutamicum* was estimated to account for approximately 20% of the total protein. Furthermore, the transcriptome and proteome of the hemoglobin overproducing strain were profiled and potential gene targets affecting hemoglobin expression were screened. This study provides a promising route for the sustainable production of hemoglobin and other recombinant proteins.

## Materials and methods

### Chemicals and reagents

All chemicals were purchased from Sangon Biotech (Shanghai, China) and Solarbio Life Science (Beijing, China) unless otherwise specified. Restriction enzymes were purchased from New England Biolabs (Beijing) (Beijing, China). DNA gel purification kit and plasmid extraction kit were purchased from Tiangen Biotech (Beijing, China). TransStart® FastPfu DNA Polymerase and T4 DNA ligase were purchased from TransGen Biotech (Beijing, China). ClonExpress MultiS one step cloning kit was purchased from Vazyme (Nanjing, China). Oligonucleotides and genes were synthesized by GENEWIZ (Suzhou, China).

### Bacterial strains and cultivation conditions

The bacterial strains used in this study are listed in Additional file 1: Table S5. *E. coli* DH5α was used for general cloning and cultivated aerobically at 37 °C in Luria–Bertani (LB) broth. Chloramphenicol (Cm, 20 μg/mL) and kanamycin (Km, 50 μg/mL) were added as required. *C. glutamicum* ATCC 13032 and its derivatives were cultivated aerobically at 30 °C in TSB medium (glucose, 5 g/L; yeast extract, 5 g/L; soy peptone, 9 g/L; urea, 3 g/L; succinic acid, 0.5 g/L; K_2_HPO_4_·3H_2_O, 1 g/L; MgSO_4_·7H2O, 0.1 g/L; biotin, 0.01 mg/L; vitamin B1, 0.1 mg/L; MOPS, 20 g/L) for maintenance and propagation. To express hemoglobin proteins in 96-well plates, recombinant strains were inoculated into 300 μL of LB medium supplemented with 10 g/L glucose, 1 g/L ALA, and 0.2 g/L FeSO_4_·7H_2_O to prevent iron and heme deficiency. When the optical density at 600 nm (OD_600nm_) of the culture reached 0.5–0.6, 0.1 mM IPTG, 100 mM sodium gluconate, or 1 g/L sodium propionate was added for inducing protein expression. To express hemoglobin proteins in shake flasks, recombinant strains were inoculated into 20 mL medium in 100 mL shake flasks. The same medium and induction condition as the fermentation in 96-well plates were used.

### Plasmid and library construction

The plasmids used in this study and the primers used for plasmid and library construction were listed in Additional file 1: Tables S5 and S6, respectively. All plasmids were constructed by homologous recombination assembly using the ClonExpress MultiS one step cloning kit (Vazyme, Nanjing, China). The hemoglobin expression plasmids were constructed based on the plasmid pXMJ19 [33]. The coding sequences of hemoglobin from different sources were optimized according to the codon usage bias of *C. glutamicum* and *E. coli* and synthesized by GENEWIZ (Suzhou, China). The construction of plasmids and libraries and the primers used are described in detail in Additional file 1: Table S6. All constructed plasmids were verified by colony PCR and Sanger sequencing.

### Gene deletion in *C. glutamicum*

To construct the ∆*gntK* mutant, plasmid pK18∆*gntK* was constructed. The left and right homologous fragments for deleting *gntK* were amplified from the genomic DNA of *C. glutamicum* ATCC 13032 by PCR. Deletion of *gntK* was achieved by two rounds of homologous recombination as described previously [47]. To construct the *∆prpDBC2* mutant, plasmid pCas9gRNA–∆*prpDBC2* was constructed and the chromosomal *prpDBC2* gene was knocked out in the strain ATCC 13032 using the CRISPR/Cas9 system as described previously [22, 48]. The construction of plasmids pK18∆*gntK* and pCas9gRNA–∆*prpDBC2* are described in detail in Additional file 1: Table S6. All constructed plasmids and gene-deleted mutants were verified by colony PCR and Sanger sequencing.

### Analytical methods

Cell biomass was determined by the OD_600nm_ with a UV-1800 spectrophotometer (Shimadzu, Kyoto, Japan) after proper dilution with distilled water. Extracellular heme was determined using the Heme Assay Kit (Catalog Number MAK316, Sigma-Aldrich Co., USA). Fluorescence intensities of hemoglobin–GFP fusion were determined using a microplate reader (SpectraMax M5, Molecular Devices, λ excitation = 488 nm, λ emission = 525 nm). The fluorescence intensities were normalized with OD_600nm_. For analysis of hemoglobin expression by SDS–PAGE, cells were collected by centrifugation, washed twice, and resuspended in PBS buffer (2.69 g/L KH_2_PO_4_, 18.24 g/L K_2_HPO_4_·3H_2_O). Cells were lysed using a high-pressure homogenizer at 4 °C. The cell lysate was centrifuged at 4 °C and 6000×*g* for 30 min. The total protein concentration in the supernatant was determined by the Bradford method. Soluble protein samples with normalized total protein concentration were used for SDS–PAGE. The ImageJ software (NIH, USA) and the Gel-Pro Analyzer software (Media Cybernetics, USA), which have been used for estimation of protein expression levels based on gel electrophoresis [49, 50], were used to estimate the expression levels of hemoglobin in this study. The heme-binding ratio of the produced hemoglobin was determined using the following equation. The heme concentrations of cell lysate of the strain overexpressing Lba (*C*_heme-Lba_) and the control strain with an empty plasmid (*C*_heme-control_) were determined using the Heme Assay Kit (Catalog Number MAK316, Sigma-Aldrich Co., USA). The concentration of total protein of the cell lysate (*C*_total protein_) was determine with BCA protein assay kit (Thermo Fisher Scientific, USA). The ratio of produced hemoglobin in the total protein (*R*_Lba_) was estimated using the ImageJ software (NIH, USA) and the Gel-Pro Analyzer software (Media Cybernetics, USA) based on the SDS–PAGE analysis:

$${R}_{\mathrm{heme}}=\frac{{C}_{\mathrm{heme}-Lba}-{C}_{\mathrm{heme}-\mathrm{control}}}{{C}_{total protein}\times {R}_{Lba}\div {Mw}_{Lba}}$$

### Omics analysis

The *C. glutamicum* strain overexpressing hemoglobin Lba-Ec (harboring pXMJ19-P_*prpD2*_-Lba-P6N1-*copA1*) and a control strain harboring the empty plasmid pXMJ19 were cultivated in LB medium supplemented with 10 g/L glucose, 1 g/L ALA, and 0.2 g/L FeSO_4_·7H_2_O. When the OD_600nm_ of the culture reached 0.5–0.6, 1 g/L sodium propionate was added for inducing protein expression. Cells at the late-exponential phase were collected by centrifugation at 6000×*g* for 5 min and immediately frozen in liquid nitrogen. Three biological replicates were conducted. Transcriptomic and proteomics analyses was performed by Novogene Co., Ltd (Tianjin, China) using the stored cell samples. For transcriptomic analysis, total RNAs were extracted and the rRNAs were removed. RNA samples were sequenced using the Illumina Hiseq X-Ten platform. Differentially expressed genes were analyzed using the DESeq2 package (v1.18.1) [51]. |log2(Fold Change)|> 1 and *p* < 0.05 were used as the criteria for identifying the differentially expressed genes. To analyze the differentially expressed genes at the functional level, KEGG clustering analysis was performed using the DAVID database as previously described [52].

For proteomics analysis, total protein was extracted and dissolved by dissolution buffer (8 M Urea, 100 mM TEAB, pH 8.5). Each protein sample was labeled by TMT [53] and fractionated using a C18 column (Waters BEH C18, 4.6 × 250 mm, 5 μm) on a Rigol L3000 HPLC. The separated peptides were analyzed by Orbitrap Exploris 480 matched with FAIMS (Thermo Fisher, USA), with ion source of Nanospray Flex™ (ESI), spray voltage of 2.1 kV, and ion transport capillary temperature of 320 ℃. The resulting spectra from each run were searched separately against the protein data of *C. glutamicum* ATCC 13032 by the search engines: Proteome Discoverer 2.4 (Thermo Fisher, USA). The protein quantitation results were statistically analyzed by Student’s *t* test. The proteins whose quantitation significantly different between experimental and control groups (|log2(Fold Change)|> 1, *p* < 0.05) were defined as differentially expressed proteins.

## Supplementary Information

**Additional file 1: Figure S1.** Extracellular accumulation of heme caused by supplement of ALA and FeSO_4_. **Figure S2.** Expression of the native hemoglobin genes without codon optimization in *C. glutamicum*. **Figure S3.** Linear relationship between the hemoglobin proportion of the total protein and GFP fluorescence/OD_600_. **Table S1.** Gene sequences used in this study. **Table S2.** NCS variants screened in this study. **Table S3.** Promoter variants screened in this study. **Table S5.** Strains and plasmids used in this study**. Table S6.** Primers used for plasmid and library construction in this study.

**Additional file 2: Table S4.** Differentially expressed genes caused by overproduction of hemoglobin Lba-Ec.

## Data Availability

The data sets used and analyzed during the current study are available from the corresponding author on reasonable request.

## References

[1] Xu X, Sharma P, Shu S, Lin T-S, Ciais P, Tubiello FN, Smith P, Campbell N, Jain AK (2021). Global greenhouse gas emissions from animal-based foods are twice those of plant-based foods. Nat Food.

[2] Kumar P, Chatli M, Mehta N, Singh P, Malav O, Verma AK (2017). Meat analogues: health promising sustainable meat substitutes. Crit Rev Food Sci Nutr.

[3] Simsa R, Yuen J, Stout A, Rubio N, Fogelstrand P, Kaplan DL (2019). Extracellular heme proteins influence bovine myosatellite cell proliferation and the color of cell-based meat. Foods.

[4] Fraser RZ, Shitut M, Agrawal P, Mendes O, Klapholz S (2018). Safety evaluation of soy leghemoglobin protein preparation derived from *Pichia pastoris*, intended for use as a flavor catalyst in plant-based meat. Int J Toxicol.

[5] Suman SP, Joseph P (2013). Myoglobin chemistry and meat color. Annu Rev Food Sci Technol.

[6] Zhao X, Zhou J, Du G, Chen J (2021). Recent advances in the microbial synthesis of hemoglobin. Trends Biotechnol.

[7] Vázquez-Limón C, Hoogewijs D, Vinogradov SN, Arredondo-Peter R (2012). The evolution of land plant hemoglobins. Plant Sci.

[8] Varnado CL, Mollan TL, Birukou I, Smith BJ, Henderson DP, Olson JS (2013). Development of recombinant hemoglobin-based oxygen carriers. Antioxid Redox Signal.

[9] Villarreal D, Phillips C, Kelley A, Villarreal S, Villaloboz A, Hernandez P, Olson J, Henderson D (2008). Enhancement of recombinant hemoglobin production in *Escherichia coli* BL21 (DE3) containing the *Plesiomonas shigelloides* heme transport system. Appl Environ Microbiol.

[10] Weickert MJ, Pagratis M, Glascock CB, Blackmore R (1999). A mutation that improves soluble recombinant hemoglobin accumulation in *Escherichia coli* in heme excess. Appl Environ Microbiol.

[11] Smith B, Gutierrez P, Guerrero E, Brewer C, Henderson D (2011). Development of a method to produce hemoglobin in a bioreactor culture of *Escherichia coli* BL21 (DE3) transformed with a plasmid containing *Plesiomonas shigelloides* heme transport genes and modified human hemoglobin genes. Appl Environ Microbiol.

[12] Shankar S, Hoyt M. Expression constructs and methods of genetically engineering methylotrophic yeast. Impossible Foods Inc. 2016, US10689656B2.

[13] Zhang B, Zhao X, Wang Z, Wang H, Zhou J, Du G, Chen J, Li J (2021). Efficient secretory expression and purification of food-grade porcine myoglobin in *Komagataella phaffii*. J Agric Food Chem.

[14] Shao Y, Xue C, Liu W, Zuo S, Wei P, Huang L, Lian J, Xu Z (2022). High-level secretory production of leghemoglobin in *Pichia pastoris* through enhanced globin expression and heme biosynthesis. Bioresour Technol.

[15] Liu L, Martínez JL, Liu Z, Petranovic D, Nielsen J (2014). Balanced globin protein expression and heme biosynthesis improve production of human hemoglobin in *Saccharomyces cerevisiae*. Metab Eng.

[16] Ishchuk OP, Frost AT, Muñiz-Paredes F, Matsumoto S, Laforge N, Eriksson NL, Martínez JL, Petranovic D (2021). Improved production of human hemoglobin in yeast by engineering hemoglobin degradation. Metab Eng.

[17] Xue J, Zhou J, Li J, Du G, Chen J, Wang M, Zhao X (2023). Systematic engineering of *Saccharomyces cerevisiae* for efficient synthesis of hemoglobins and myoglobins. Bioresour Technol.

[18] Wolf S, Becker J, Tsuge Y, Kawaguchi H, Kondo A, Marienhagen J, Bott M, Wendisch VF, Wittmann C (2021). Advances in metabolic engineering of *Corynebacterium glutamicum* to produce high-value active ingredients for food, feed, human health, and well-being. Essays Biochem.

[19] Chai M, Deng C, Chen Q, Lu W, Liu Y, Li J, Du G, Lv X, Liu L (2021). Synthetic biology toolkits and metabolic engineering applied in *Corynebacterium glutamicum* for biomanufacturing. ACS Synth Biol.

[20] Cho JS, Choi KR, Prabowo CPS, Shin JH, Yang D, Jang J, Lee SY (2017). CRISPR/Cas9-coupled recombineering for metabolic engineering of *Corynebacterium glutamicum*. Metab Eng.

[21] Wang Y, Liu Y, Liu J, Guo Y, Fan L, Ni X, Zheng X, Wang M, Zheng P, Sun J (2018). MACBETH: multiplex automated *Corynebacterium glutamicum* base editing method. Metab Eng.

[22] Liu J, Liu M, Shi T, Sun G, Gao N, Zhao X, Guo X, Ni X, Yuan Q, Feng J, Liu Z, Guo Y, Chen J, Wang Y, Zheng P, Sun J (2022). CRISPR-assisted rational flux-tuning and arrayed CRISPRi screening of an L-proline exporter for L-proline hyperproduction. Nat Commun.

[23] Wang Y, Cheng H, Liu Y, Liu Y, Wen X, Zhang K, Ni X, Gao N, Fan L, Zhang Z, Liu J, Chen J, Wang L, Guo Y, Zheng P, Wang M, Sun J, Ma Y (2021). *In-situ* generation of large numbers of genetic combinations for metabolic reprogramming via CRISPR-guided base editing. Nat Commun.

[24] Liu X, Zhang W, Zhao Z, Dai X, Yang Y, Bai Z (2017). Protein secretion in *Corynebacterium glutamicum*. Crit Rev Biotechnol.

[25] Liu X, Yang Y, Zhang W, Sun Y, Peng F, Jeffrey L, Harvey L, McNeil B, Bai Z (2016). Expression of recombinant protein using *Corynebacterium glutamicum*: progress, challenges and applications. Crit Rev Biotechnol.

[26] Yim SS, Choi JW, Lee RJ, Lee YJ, Lee SH, Kim SY, Jeong KJ (2016). Development of a new platform for secretory production of recombinant proteins in *Corynebacterium glutamicum*. Biotechnol Bioeng.

[27] Jin Q, Pan F, Hu C-F, Lee SY, Xia X-X, Qian Z-G (2022). Secretory production of spider silk proteins in metabolically engineered *Corynebacterium glutamicum* for spinning into tough fibers. Metab Eng.

[28] Chen J, Wang Y, Guo X, Rao D, Zhou W, Zheng P, Sun J, Ma Y (2020). Efficient bioproduction of 5-aminolevulinic acid, a promising biostimulant and nutrient, from renewable bioresources by engineered *Corynebacterium glutamicum*. Biotechnol Biofuels.

[29] Ko YJ, Kim M, You SK, Shin SK, Chang J, Choi HJ, Jeong W-Y, Lee M-E, Hwang D-H, Han SO (2021). Animal-free heme production for artificial meat in *Corynebacterium glutamicum* via systems metabolic and membrane engineering. Metab Eng.

[30] Álvarez-Salgado E, Arredondo-Peter R (2015). Effect of the synthesis of rice non-symbiotic hemoglobins 1 and 2 in the recombinant *Escherichia coli* TB1 growth. F1000Res.

[31] Aréchaga-Ocampo E, Saenz-Rivera J, Sarath G, Klucas RV, Arredondo-Peter R (2001). Cloning and expression analysis of hemoglobin genes from maize (*Zea mays* ssp. *mays*) and teosinte (*Zea mays* ssp. *parviglumis*). Biochim Biophys Acta.

[32] Buisson N, Labbe-Bois R (1998). Flavohemoglobin expression and function in *Saccharomyces cerevisiae*. No relationship with respiration and complex response to oxidative stress. J Biol Chem.

[33] Jakoby M, Ngouoto-Nkili C-E, Burkovski A (1999). Construction and application of new *Corynebacterium glutamicum* vectors. Biotechnol Tech.

[34] Frunzke J, Gätgens C, Brocker M, Bott M (2011). Control of heme homeostasis in *Corynebacterium glutamicum* by the two-component system HrrSA. J Bacteriol.

[35] Al-Hawash AB, Zhang X, Ma F (2017). Strategies of codon optimization for high-level heterologous protein expression in microbial expression systems. Gene Rep.

[36] Wang C, Zhang W, Tian R, Zhang J, Zhang L, Deng Z, Lv X, Li J, Liu L, Du G (2022). Model-driven design of synthetic N-terminal coding sequences for regulating gene expression in yeast and bacteria. Biotechnol J.

[37] Plassmeier JK, Busche T, Molck S, Persicke M, Pühler A, Rückert C, Kalinowski J (2013). A propionate-inducible expression system based on the *Corynebacterium glutamicum prpD2* promoter and PrpR activator and its application for the redirection of amino acid biosynthesis pathways. J Biotechnol.

[38] Wiechert J, Gätgens C, Wirtz A, Frunzke J (2020). Inducible expression systems based on xenogeneic silencing and counter-silencing and design of a metabolic toggle switch. ACS Synth Biol.

[39] Frunzke J, Engels V, Hasenbein S, Gätgens C, Bott M (2008). Co-ordinated regulation of gluconate catabolism and glucose uptake in *Corynebacterium glutamicum* by two functionally equivalent transcriptional regulators, GntR1 and GntR2. Mol Microbiol.

[40] Hashiro S, Mitsuhashi M, Yasueda H (2019). High copy number mutants derived from *Corynebacterium glutamicum* cryptic plasmid pAM330 and copy number control. J Biosci Bioeng.

[41] Kirchner O, Tauch A (2003). Tools for genetic engineering in the amino acid-producing bacterium *Corynebacterium glutamicum*. J Biotechnol.

[42] Liu Y, Yang Q, Zhao F (2021). Synonymous but not silent: the codon usage code for gene expression and protein folding. Annu Rev Biochem.

[43] Boël G, Letso R, Neely H, Price WN, Wong K-H, Su M, Luff JD, Valecha M, Everett JK, Acton TB (2016). Codon influence on protein expression in *E. coli* correlates with mRNA levels. Nature.

[44] Presnyak V, Alhusaini N, Chen Y-H, Martin S, Morris N, Kline N, Olson S, Weinberg D, Baker KE, Graveley BR (2015). Codon optimality is a major determinant of mRNA stability. Cell.

[45] Beckers G, Bendt AK, Krämer R, Burkovski A (2004). Molecular identification of the urea uptake system and transcriptional analysis of urea transporter- and urease-encoding genes in *Corynebacterium glutamicum*. J Bacteriol.

[46] Yu X, Li S, Feng H, Liao X, Xing X-H, Bai Z, Liu X, Zhang C (2023). CRISPRi-microfluidics screening enables genome-scale target identification for high-titer protein production and secretion. Metab Eng.

[47] Schäfer A, Tauch A, Jäger W, Kalinowski J, Thierbach G, Pühler A (1994). Small mobilizable multi-purpose cloning vectors derived from the *Escherichia coli* plasmids pK18 and pK19: selection of defined deletions in the chromosome of *Corynebacterium glutamicum*. Gene.

[48] Liu J, Wang Y, Lu Y, Zheng P, Sun J, Ma Y (2017). Development of a CRISPR/Cas9 genome editing toolbox for *Corynebacterium glutamicum*. Microb Cell Fact.

[49] Carvajal-Vergara X, Sevilla A, D'Souza SL, Ang YS, Schaniel C, Lee DF, Yang L, Kaplan AD, Adler ED, Rozov R, Ge Y, Cohen N, Edelmann LJ, Chang B, Waghray A, Su J, Pardo S, Lichtenbelt KD, Tartaglia M, Gelb BD, Lemischka IR (2010). Patient-specific induced pluripotent stem-cell-derived models of LEOPARD syndrome. Nature.

[50] Gong Z, Wan H, Tay TL, Wang H, Chen M, Yan T (2003). Development of transgenic fish for ornamental and bioreactor by strong expression of fluorescent proteins in the skeletal muscle. Biochem Biophys Res Commun.

[51] Wang L, Feng Z, Wang X, Wang X, Zhang X (2010). DEGseq: an R package for identifying differentially expressed genes from RNA-seq data. Bioinformatics.

[52] Ju Q, Zhao YJ, Dong Y, Cheng C, Zhang S, Yang Y, Li P, Ge D, Sun B (2019). Identification of a miRNA-mRNA network associated with lymph node metastasis in colorectal cancer. Oncol Lett.

[53] Zhang H, Liu T, Zhang Z, Payne SH, Zhang B, McDermott JE, Zhou J-Y, Petyuk VA, Chen L, Ray D (2016). Integrated proteogenomic characterization of human high-grade serous ovarian cancer. Cell.

