# Supplementary material for: Sustainable and high-level microbial production of plant hemoglobin in Corynebacterium glutamicum
Source: Biotechnol Biofuels Bioprod. 2023 May 11;16:80. doi: 10.1186/s13068-023-02337-9 (PMC10176901; doi:10.1186/s13068-023-02337-9)
Supplement: Supplementary file 1 — Additional file 1: Figure S1. Extracellular accumulation of heme caused by supplement of ALA and FeSO4. Figure S2. Expression of the native hemoglobin genes without codon optimization in C. glutamicum. Figure S3. Linear relationship between the hemoglobin proportion of the total protein and GFP fluorescence/OD600. Table S1. Gene sequences used in this study. Table S2. NCS variants screened in this study. Table S3. Promoter variants screened in this study. Table S5. Strains and plasmids used in this study. Table S6. Primers used for plasmid and library construction in this study. [file 13068_2023_2337_MOESM1_ESM.docx]

Additional file 1 for

Sustainable and high-level microbial production of plant hemoglobin in *Corynebacterium glutamicum*

**This file includes:**

Figure S1

Figure S2

Figure S3

Table S1

Table S2

Table S3

Table S5

Table S6

**Table S4 is provided separately in Additional file 2.**


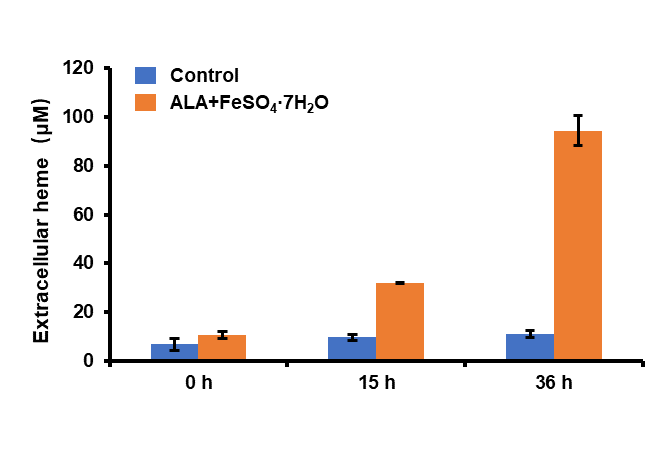


**Figure S1.** Extracellular accumulation of heme caused by supplement of ALA and FeSO_4_. *C. glutamicum* overexpressing Lba-Ec was cultivated with supplement of 1 g/L ALA, and 0.2 g/L FeSO_4_·7H_2_O. The culture without supplement of ALA and FeSO_4_·7H_2_O was used as a control. Values and error bars represent means and standard deviations (n = 3).


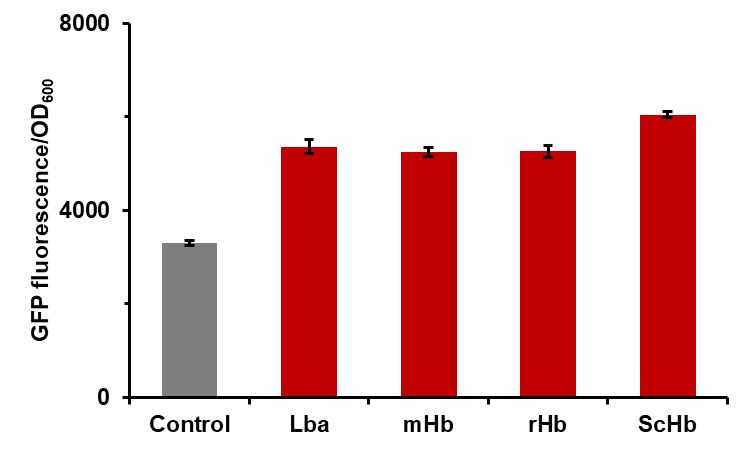


**Figure S2.** Expression of the native hemoglobin genes without codon optimization in *C. glutamicum*. The hemoglobin expression levels were characterized by measuring the fluorescence intensities of hemoglobin-GFP fusion. The strain harboring an empty plasmid was used as a control. Values and error bars represent means and standard deviations (n = 3).


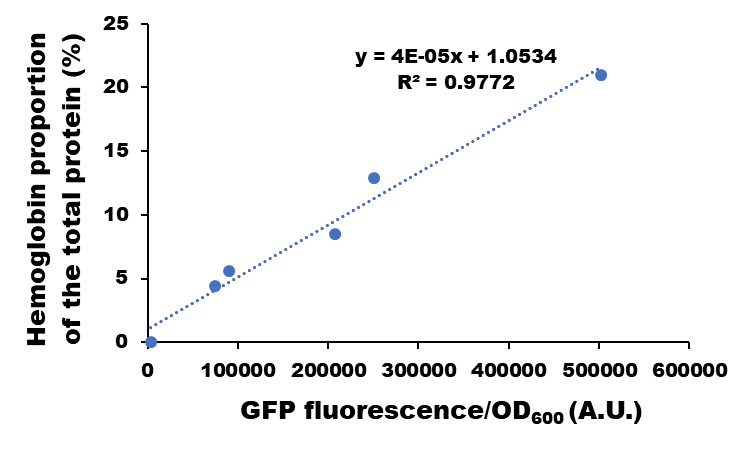


**Figure S3**. Linear relationship between the hemoglobin proportion of the total protein and GFP fluorescence/OD_600_. The hemoglobin proportion of the total protein was quantified by SDS-PAGE and the software ImageJ v1.48. The GFP fluorescence/OD_600_ was determined by measuring the fluorescence intensities of the hemoglobin-GFP fusion.

**Table S1.** Gene sequences used in this study.

| **Gene name** | **Amino acid sequence** | **Gene sequence** | **Description** |
| --- | --- | --- | --- |
| Lba | MVAFTEKQDALVSSSFEAFKANIPQYSVVFYTSILEKAPAAKDLFSFLANGVDPTNPKLTGHAEKLFALVRDSAGQLKASGTVVADAALGSVHAQKAVTDPQFVVVKEALLKTIKAAVGDKWSDELSRAWEVAYDELAAAIKKA | ATGGTTGCTTTCACTGAGAAGCAAGATGCTTTGGTGAGTAGCTCATTCGAAGCATTCAAGGCAAACATTCCTCAATACAGCGTTGTGTTCTACACTTCGATACTGGAGAAAGCACCTGCAGCAAAGGACTTGTTCTCATTTCTAGCAAATGGAGTAGACCCCACTAATCCTAAGCTCACGGGCCATGCTGAAAAGCTTTTTGCATTGGTGCGTGACTCAGCTGGTCAACTTAAAGCAAGTGGAACAGTGGTGGCTGATGCCGCACTTGGTTCTGTTCATGCCCAAAAAGCAGTCACTGATCCTCAGTTCGTGGTGGTTAAAGAAGCACTGCTGAAAACAATAAAGGCAGCAGTTGGGGACAAATGGAGTGACGAGTTGAGCCGTGCTTGGGAAGTAGCCTACGATGAATTGGCAGCAGCTATTAAGAAGGCA | The native gene of the soybean hemoglobin |
| Lba-Ec |  | ATGGTGGCGTTTACCGAAAAACAAGATGCGCTGGTGAGCAGTAGCTTTGAAGCGTTTAAAGCGAACATTCCGCAGTATAGCGTGGTGTTTTATACGAGCATTCTGGAAAAAGCGCCGGCGGCGAAAGATCTGTTTAGCTTTCTGGCGAACGGCGTGGATCCGACCAACCCGAAACTGACCGGCCATGCGGAAAAACTGTTTGCCCTGGTGCGCGATAGCGCGGGTCAGCTGAAAGCGAGCGGCACCGTGGTGGCGGATGCGGCGCTGGGCAGCGTGCATGCGCAGAAAGCGGTGACCGATCCGCAGTTTGTGGTTGTGAAAGAAGCGCTGCTGAAAACCATTAAAGCGGCGGTGGGCGATAAATGGAGCGATGAACTGAGCCGCGCGTGGGAAGTGGCGTATGATGAACTGGCGGCCGCGATTAAAAAAGCG | Codon optimization of soybean hemoglobin gene according to the codon usage bias of *E. coli* |
| Lba-Cg |  | ATGGTTGCTTTCACCGAGAAGCAGGACGCTCTGGTTTCCTCCTCCTTCGAGGCTTTCAAGGCTAACATCCCACAGTACTCCGTTGTTTTCTACACCTCCATCCTGGAGAAGGCTCCAGCTGCTAAGGACCTGTTCTCCTTCCTGGCTAACGGCGTTGACCCAACCAACCCAAAGCTGACCGGCCACGCTGAGAAGCTGTTCGCTCTGGTTCGCGACTCCGCTGGCCAGCTGAAGGCTTCCGGCACCGTTGTTGCTGACGCTGCTCTGGGCTCCGTTCACGCTCAGAAGGCTGTTACCGACCCACAGTTCGTTGTTGTTAAGGAGGCTCTGCTGAAGACCATCAAGGCTGCTGTTGGCGACAAGTGGTCCGACGAGCTGTCCCGCGCTTGGGAGGTTGCTTACGACGAGCTGGCTGCTGCTATCAAGAAGGCT | Codon optimization of soybean hemoglobin gene according to the codon usage bias of *C. glutamicum* |
| mHb | MALAEADDGAVVFGEEQEALVLKSWAVMKKDAANLGLRFFLKVFEIAPSAEQMFSFLRDSDVPLEKNPKLKTHAMSVFVMTCEAAAQLRKAGKVTVRETTLKRLGATHLRYGVADGHFEVTGFALLETIKEALPADMWSLEMKKAWAEAYSQLVAAIKREMKPDA | ATGGCACTCGCGGAGGCCGACGACGGCGCGGTGGTCTTCGGCGAGGAGCAGGAGGCGCTGGTGCTCAAGTCGTGGGCCGTCATGAAGAAGGACGCCGCAAACCTGGGCCTCCGCTTCTTCCTCAAGGTCTTCGAGATCGCGCCGTCGGCGGAGCAGATGTTCTCGTTCCTGCGCGACTCCGACGTGCCGCTAGAGAAGAACCCCAAGCTCAAGACGCACGCCATGTCCGTCTTCGTCATGACCTGCGAGGCGGCGGCGCAGCTTCGCAAGGCCGGGAAGGTCACCGTGAGGGAGACCACGCTCAAGAGGCTGGGCGCCACGCACTTGAGGTACGGCGTCGCAGATGGACACTTCGAGGTGACGGGGTTCGCGCTGCTTGAGACGATCAAGGAGGCGCTCCCCGCTGACATGTGGAGCCTCGAGATGAAGAAAGCCTGGGCCGAGGCCTACAGCCAGCTGGTGGCGGCCATCAAGCGGGAGATGAAGCCCGATGCC | The native gene of the maize hemoglobin |
| mHb-Ec |  | ATGGCGCTGGCGGAAGCGGATGATGGCGCGGTGGTGTTTGGCGAAGAACAAGAAGCGCTGGTGCTGAAAAGCTGGGCGGTGATGAAAAAAGATGCGGCGAACCTGGGCCTGCGCTTTTTTCTGAAAGTGTTTGAAATTGCGCCGAGCGCGGAACAGATGTTTAGCTTTCTGCGCGATAGCGATGTGCCGCTGGAAAAAAACCCGAAACTGAAAACCCATGCGATGAGCGTGTTTGTGATGACCTGCGAAGCGGCCGCGCAGCTGCGCAAAGCGGGCAAAGTGACCGTGCGCGAAACCACCCTGAAACGCCTGGGCGCGACCCATCTGCGCTATGGCGTGGCGGATGGCCATTTTGAAGTGACCGGCTTTGCGCTGCTGGAAACCATTAAAGAAGCGCTGCCGGCGGATATGTGGAGCCTGGAAATGAAAAAAGCGTGGGCGGAAGCGTATAGTCAGCTGGTGGCGGCGATTAAACGCGAAATGAAACCGGATGCG | Codon optimization of maize hemoglobin gene according to the codon usage bias of *E. coli* |
| mHb-Cg |  | ATGGCACTGGCAGAAGCAGATGATGGCGCAGTGGTGTTCGGCGAAGAACAAGAAGCACTGGTGCTGAAGTCCTGGGCAGTGATGAAGAAGGATGCAGCAAACCTGGGCCTGCGCTTCTTCCTGAAGGTGTTCGAAATCGCACCATCCGCAGAACAGATGTTCTCCTTCCTGCGCGATTCCGATGTGCCACTGGAAAAGAACCCAAAGCTGAAGACCCACGCAATGTCCGTGTTCGTGATGACCTGCGAAGCAGCCGCACAGCTGCGCAAGGCCGGCAAGGTGACCGTGCGCGAAACCACCCTGAAGCGCCTGGGCGCAACCCACCTGCGCTACGGCGTGGCAGATGGCCACTTCGAAGTGACCGGCTTCGCACTGCTGGAAACCATCAAGGAAGCACTGCCAGCAGATATGTGGTCCCTGGAAATGAAGAAGGCATGGGCAGAAGCATACTCTCAGCTGGTGGCAGCAATCAAGCGCGAAATGAAGCCTGATGCA | Codon optimization of maize hemoglobin gene according to the codon usage bias of *C. glutamicum* |
| rHb | MAANGSNVVSRGAVRFTEEQEALVLKSWAIMKNDSAHIGHRFFLKIFEVAPSARQLFSFLRNSDVPLEKNPKLKIHAMAVFVMTCEAAAQLRKTGRVTVRDTTIKRLGSTHFKNGVSDAHFEVAKFALLETIKEAVPASMWSPAMKGAWGEAYDHLVAAIKQGMKPAAA | ATGGCTGCGAACGGAAGCAACGTCGTGTCGAGGGGAGCAGTCAGGTTCACCGAGGAGCAGGAGGCGCTGGTGCTCAAGTCGTGGGCCATCATGAAGAACGACTCCGCCCACATTGGGCACCGCTTCTTCCTTAAGATCTTCGAGGTCGCGCCGTCGGCGCGCCAGCTGTTCTCGTTCCTGCGAAACTCCGACGTGCCGCTCGAGAAGAACCCCAAGCTCAAGATCCACGCCATGGCCGTCTTCGTCATGACGTGCGAGGCGGCTGCGCAGCTGCGGAAAACCGGGAGGGTCACCGTGAGGGACACCACCATCAAGAGGCTGGGCTCCACGCACTTCAAGAACGGCGTCTCCGACGCCCACTTCGAGGTGGCGAAGTTCGCGCTGCTTGAGACGATCAAGGAGGCGGTTCCGGCGAGCATGTGGAGCCCCGCGATGAAGGGCGCGTGGGGCGAAGCTTACGACCACCTGGTTGCAGCCATCAAGCAGGGGATGAAGCCGGCTGCTGCG | The native gene of the rice hemoglobin |
| rHb-Ec |  | ATGGCGGCGAACGGCAGCAACGTGGTGAGCCGCGGCGCGGTGCGCTTTACCGAAGAACAAGAAGCGCTGGTGCTGAAAAGCTGGGCGATTATGAAAAACGATAGCGCGCATATTGGCCATCGCTTTTTTCTGAAAATTTTTGAGGTGGCGCCGAGCGCGCGTCAGCTGTTTAGCTTTCTGCGCAACAGCGATGTGCCGCTGGAAAAAAACCCGAAACTGAAAATTCATGCGATGGCGGTGTTTGTGATGACCTGCGAAGCGGCCGCGCAGCTGCGCAAAACCGGCCGCGTGACCGTGCGCGATACCACCATTAAACGCCTGGGCAGCACCCATTTTAAAAACGGCGTGAGCGATGCGCATTTTGAAGTGGCGAAATTTGCGCTGCTGGAAACCATTAAAGAAGCGGTGCCGGCGAGCATGTGGAGCCCGGCGATGAAAGGCGCGTGGGGCGAAGCGTATGATCATCTGGTGGCGGCGATTAAACAAGGCATGAAACCGGCGGCCGCG | Codon optimization of rice hemoglobin gene according to the codon usage bias of *E. coli* |
| rHb-Cg |  | ATGGCAGCAAACGGCTCCAACGTGGTGTCCCGCGGCGCAGTGCGCTTCACCGAAGAACAAGAAGCACTGGTGCTGAAGTCCTGGGCAATCATGAAGAACGATTCCGCACACATCGGCCACCGCTTCTTCCTGAAGATCTTCGAGGTGGCACCATCCGCACGTCAGCTGTTCTCCTTCCTGCGCAACTCCGATGTGCCACTGGAAAAGAACCCAAAGCTGAAGATCCACGCAATGGCAGTGTTCGTGATGACCTGCGAAGCAGCCGCACAGCTGCGCAAGACCGGCCGCGTGACCGTGCGCGATACCACCATCAAGCGCCTGGGCTCCACCCACTTCAAGAACGGCGTGTCCGATGCACACTTCGAAGTGGCAAAGTTCGCACTGCTGGAAACCATCAAGGAAGCAGTGCCTGCATCCATGTGGTCCCCTGCAATGAAGGGCGCATGGGGCGAAGCATACGATCACCTGGTGGCAGCAATCAAGCAAGGCATGAAGCCAGCAGCCGCA | Codon optimization of rice hemoglobin gene according to the codon usage bias of *C. glutamicum* |
| ScHb | MLAEKTRSIIKATVPVLEQQGTVITRTFYKNMLTEHTELLNIFNRTNQKVGAQPNALATTVLAAAKNIDDLSVLMDHVKQIGHKHRALQIKPEHYPIVGEYLLKAIKEVLGDAATPEIINAWGEAYQAIADIFITVEK | ATGCTAGCCGAAAAAACCCGTTCCATAATCAAAGCAACCGTTCCTGTTTTGGAACAGCAGGGCACTGTCATCACGCGTACATTCTACAAAAATATGCTTACTGAACACACCGAATTACTTAATATTTTTAACAGAACTAATCAAAAGGTTGGCGCACAACCAAATGCCTTGGCCACTACTGTTTTGGCAGCGGCTAAGAACATTGACGATTTGTCTGTGCTTATGGACCATGTCAAGCAAATTGGCCACAAGCATCGTGCCTTGCAAATTAAACCTGAGCATTATCCAATCGTTGGTGAGTACTTGTTAAAAGCAATCAAGGAAGTTTTAGGCGATGCTGCGACTCCAGAGATAATTAATGCATGGGGCGAGGCTTACCAAGCAATTGCTGACATCTTCATCACTGTTGAAAAA | The native gene of the yeast hemoglobin |
| ScHb-Ec |  | ATGCTGGCGGAAAAAACCCGCAGCATTATTAAAGCGACCGTGCCGGTGCTGGAACAGCAAGGCACCGTGATTACCCGCACCTTTTATAAAAACATGCTGACCGAACATACCGAACTGCTGAACATTTTTAACCGCACCAATCAGAAAGTGGGCGCGCAGCCGAACGCGCTGGCGACCACCGTGCTGGCGGCCGCGAAAAACATTGATGATCTGAGCGTGCTGATGGATCATGTGAAACAGATTGGCCATAAACATCGCGCGTTACAGATTAAACCGGAACATTATCCGATTGTGGGCGAATATCTGCTGAAAGCGATTAAAGAAGTGCTGGGCGATGCGGCGACCCCGGAAATTATTAACGCGTGGGGCGAAGCGTATCAAGCGATTGCGGATATTTTTATTACCGTGGAAAAA | Codon optimization of yeast hemoglobin gene according to the codon usage bias of *E. coli* |
| ScHb-Cg |  | ATGCTGGCAGAAAAGACCCGCTCCATCATCAAGGCAACCGTGCCTGTGCTGGAACAGCAAGGCACCGTGATCACCCGCACCTTCTACAAGAACATGCTGACCGAACACACCGAACTGCTGAACATCTTCAACCGCACCAATCAGAAGGTGGGCGCACAGCCAAACGCACTGGCAACCACCGTGCTGGCAGCCGCAAAGAACATCGATGATCTGTCCGTGCTGATGGATCACGTGAAGCAGATCGGCCACAAGCACCGCGCACTGCAAATCAAGCCTGAACACTACCCAATCGTGGGCGAATACCTGCTGAAGGCAATCAAGGAAGTGCTGGGCGATGCAGCAACCCCTGAAATCATCAACGCATGGGGCGAAGCATACCAAGCAATCGCAGATATCTTCATCACCGTGGAAAAG | Codon optimization of yeast hemoglobin gene according to the codon usage bias of *C. glutamicum* |
| P*_prpD2_* | - | CTCCAGCGTCCAAGAATATGCCCCCGCGCGCCGGGTGGGGAGCGAAGGGAACCCCCAAGGAATTGGCGTTGAGGTGGTGATTTTGCATGTTTTACTCAAAATCACTTTGATGGTCACAAAATTACACAACTTTTACAGTGACCTACATTGCTTTTTAAAGAATTAGTGTGGTGTGC | The original sequence of P*_prpD2_* |
| P*_gntk_* | - | GTATCAATGGAATCCGGGACGCGATGAAAACTATGACCTCGTGATCAACACCGGTTCGATGACATACGAACAAATCGTTGATCTAGTTGTGGAAACTTACGCCAGGAAGTATCCGCTCCACGTGAGAATCATTCCGAACGGAAAAGACCAATAAACATACAGTCCCCGTGATGTGACCATACACACCACGGGGACTGTGGCGTAGGTCTTACAAAATTCCCCAAAAAGAGTTATGATAGTACCAATAAGTTTTTGTGGCAGCCTCCTGCATTCGGCAGTCGAGACGCCACCAAAGAAAGGATAAGAC | The original sequence of P*_gntK_* |

**Table S2.** NCS variants screened in this study.

| **NCS** | **Amino acid sequence** | **Nucleotide sequence** |
| --- | --- | --- |
| Lba-Ec | MVAFTEKQDALV | ATGGTGGCGTTTACCGAAAAACAAGATGCGCTGGTG |
| Lba#N1 |  | ATGGTTGCATTTACAGAAAAACAAGACGCCCTAGTT |
| Lba#N2 |  | ATGGTGGCTTTCACTGAGAAACAGGACGCGCTCGTC |
| Lba#N3 |  | ATGGTAGCGTTTACAGAGAAACAAGATGCACTTGTC |
| Lba#N4 |  | ATGGTAGCGTTTACTGAAAAACAAGATGCGCTCGTT |
| mHb-Cg | MALAEADDGAVV | ATGGCACTGGCAGAAGCAGATGATGGCGCAGTGGTG |
| mHb#N1 |  | ATGGCCCTTGCCGAGGCCGATGACGGGGCTGTAGTG |
| mHb#N2 |  | ATGGCCCTTGCTGAGGCTGACGATGGTGCTGTGGTA |
| mHb#N3 |  | ATGGCTCTTGCAGAAGCCGATGACGGCGCTGTGGTT |
| mHb#N4 |  | ATGGCACTGGCTGAAGCTGATGATGGCGCCGTGGTT |

**Table S3.** Promoter variants screened in this study.

| **Promoter name** | **Sequence of the mutated region** | **Description** |
| --- | --- | --- |
| PprpD2 | TTGCTTTTTAAAGAATTAGTG | Original sequence |
| Lba-PprpD2#1 | TTGTCGTGCCGGCTGCTAGAC | Sequence of the screened promoter variants |
| Lba-PprpD2#2 | CTAGCTGATGTGTGATATGG |  |
| Lba-PprpD2#3 | ATATGCGGGGATAGTGTAGAA |  |
| Lba-PprpD2#4 | TTAGGATTATCTAGGTTAAGA |  |
| Lba-PprpD2#5 | GGAAATTACTTATGATATAG |  |
| Lba-PprpD2#6 | GTCTTTCTTGTGTTATAATT |  |
| Lba-PprpD2#7 | GTGTCTATTAGTATGGTATAG |  |
| Lba-PprpD2#8 | GGGCGCCTACTATGGTACGG |  |
| Lba-PprpD2#9 | AGCCCTATGACCGTGCTAGGA |  |
| Lba-PprpD2#10 | AAGCCGTTATATGTGGTAAGT |  |
| Lba-PprpD2#11 | ACCGGTAGAATTATGCTATGC |  |
| mHb-PprpD2#1 | TAGCCGGTTGGTGTGCTATAT |  |
| mHb-PprpD2#2 | CGTTTTTAGCCTATGTTAAGC |  |
| mHb-PprpD2#3 | CAGTCTCTAATGCTAGAA |  |
| mHb-PprpD2#4 | CATGGTGCGTATCGTGTAAGT |  |
| mHb-PprpD2#5 | TCGAGAAGATTCGTGTTAGGA |  |
| mHb-PprpD2#6 | TTTGGTTCTTCTGCTGTAACG |  |
| mHb-PprpD2#7 | GGTTAGTCTCATATGCTAGTG |  |
| mHb-PprpD2#8 | TAATTCGTCATTGTGTTATCA |  |
| mHb-PprpD2#9 | TATTTTGGTCATGTGGTAAGG |  |
| mHb-PprpD2#10 | GCGATTTAATATATGGTAGTG |  |
| mHb-PprpD2#11 | AACGTTGCCTTAATGCTATGA |  |
| mHb-PprpD2#12 | GTCGCCCCGCTTGTGATACAA |  |
| mHb-PprpD2#13 | AGTGCTCGTCCTGTGGTATGG |  |

**Table S5.** Strains and plasmids used in this study.

| **Strain** | **Description** | **Source** |
| --- | --- | --- |
| *C. glutamicum* ATCC 13032 | Wild type | Lab stock |
| *E. coli* Trans1-T1 | F-φ80(*lacZ*)Δ*M15*Δ*lacX*74*hsdR*(rk-, mk+)Δ*recA*1398*endA*1*tonA* | TransGen Biotech |
| *C. glutamicum* ATCC 13032∆*prpDBC2* | ATCC 13032 derivative with deletion of gene cluster *prpDBC2* | This study |
| *C. glutamicum* ATCC 13032∆*gntK* | ATCC 13032 derivative with deletion of gene *gntK* | This study |
| **Plasmid** |  |  |
| pXMJ19 | Expression vector of *C. glutamicum*, IPTG-inducible promoter P*_tac_*, Cm^R^ | [1] |
| pK18*mobsacB* | Suicide plasmid for gene knockout, Kan^R^ | [2] |
| pCas9gRNA-*ccdB* | Tool plasmid for gene deletion by CRISPR/Cas9 | [3] |
| pCas9gRNA-∆*prpDBC2* | pCas9gRNA-*ccdB* derivative, carrying the homology arm for *prpDBC2* deletion and the gRNA targeting *prpDBC2* | This study |
| pK18∆*gntK* | pK18*mobsacB*, carrying the homology arm for *gntK* deletion | This study |
| pXMJ19-Lba-Ec | pXMJ19 derivative harboring *Glycine max* hemoglobin A gene with codon optimization with *E. coli* genome as a reference | This study |
| pXMJ19-Lba-Cg | pXMJ19 derivative harboring *Glycine max* hemoglobin A gene with codon optimization with *C. glutamicum* genome as a reference | This study |
| pXMJ19-mHb-Ec | pXMJ19 derivative harboring *Zea mays* hemoglobin gene with codon optimization with *E. coli* genome as a reference | This study |
| pXMJ19-mHb-Cg | pXMJ19 derivative harboring *Zea mays* hemoglobin gene with codon optimization with *C. glutamicum* genome as a reference | This study |
| pXMJ19-rHb-Ec | pXMJ19 derivative harboring *Oryza sativa* hemoglobin gene with codon optimization with *E. coli* genome as a reference | This study |
| pXMJ19-rHb-Cg | pXMJ19 derivative harboring *Oryza sativa* hemoglobin gene with codon optimization with *C. glutamicum* genome as a reference | This study |
| pXMJ19-ScHb-Ec | pXMJ19 derivative harboring *S. cerevisiae* hemoglobin gene with codon optimization with *E. coli* genome as a reference | This study |
| pXMJ19-ScHb-Cg | pXMJ19 derivative harboring *S. cerevisiae* hemoglobin gene with codon optimization with *C. glutamicum* genome as a reference | This study |
| pXMJ19-Lba-*gfp* | pXMJ19 derivative harboring the native Lba gene fused to *gfp* | This study |
| pXMJ19-mHb-*gfp* | pXMJ19 derivative harboring the native mHb gene fused to *gfp* | This study |
| pXMJ19-rHb-*gfp* | pXMJ19 derivative harboring the native rHb gene fused to *gfp* | This study |
| pXMJ19-ScHb-*gfp* | pXMJ19 derivative harboring the native ScHb gene fused to *gfp* | This study |
| pXMJ19-Lba-Ec-*gfp* | pXMJ19 derivative harboring Lba-Ec-*gfp* fusion gene | This study |
| pXMJ19-Lba-Cg-*gfp* | pXMJ19 derivative harboring Lba-Cg-*gfp* fusion gene | This study |
| pXMJ19-mHb-Ec-*gfp* | pXMJ19 derivative harboring mHb-Ec-*gfp* fusion gene | This study |
| pXMJ19-mHb-Cg-*gfp* | pXMJ19 derivative harboring mHb-Cg-*gfp* fusion gene | This study |
| pXMJ19-rHb-Ec-*gfp* | pXMJ19 derivative harboring rHb-Ec-*gfp* fusion gene | This study |
| pXMJ19-rHb-Cg-*gfp* | pXMJ19 derivative harboring rHb-Cg-*gfp* fusion gene | This study |
| pXMJ19-ScHb-Ec-*gfp* | pXMJ19 derivative harboring ScHb-Ec-*gfp* fusion gene. | This study |
| pXMJ19-ScHb-Cg-*gfp* | pXMJ19 derivative harboring ScHb-Cg-*gfp* fusion gene | This study |
| pXMJ19-P*_prpD2_*-Lba-Ec-*gfp* | pXMJ19-Lba-Ec-*gfp* derivative with P*_tac_* replaced with P*_prpD2_* | This study |
| pXMJ19-P*_prpD2_*-mHb-Cg-*gfp* | pXMJ19-mHb-Cg-*gfp* derivative with P*_tac_* replaced with P*_prpD2_* | This study |
| pXMJ19-P*_gntK_*-Lba-Ec-*gfp* | pXMJ19-Lba-Ec-*gfp* derivative with P*_tac_* replaced with P*_gntK_* | This study |
| pXMJ19-P*_gntK_*-mHb-Cg-*gfp* | pXMJ19-mHb-Cg-*gfp* derivative with P*_tac_* replaced with P*_gntK_* | This study |
| pXMJ19-P*_prpD2_*-Lba-P1N1-*gfp* | pXMJ19-P*_prpD2_*-Lba-Ec-*gfp* derivative with the P*_prpD2_* promoter variant P1 combined with the NCS variant N1 | This study |
| pXMJ19-P*_prpD2_*-Lba-P1N2-*gfp* | pXMJ19-P*_prpD2_*-Lba-Ec-*gfp* derivative with the P*_prpD2_* promoter variant P1 combined with the NCS variant N2 | This study |
| pXMJ19-P*_prpD2_*-Lba-P3N1-*gfp* | pXMJ19-P*_prpD2_*-Lba-Ec-*gfp* derivative with the P*_prpD2_* promoter variant P3 combined with the NCS variant N1 | This study |
| pXMJ19-P*_prpD2_*-Lba-P3N2-*gfp* | pXMJ19-P*_prpD2_*-Lba-Ec-*gfp* derivative with the P*_prpD2_* promoter variant P3 combined with the NCS variant N2 | This study |
| pXMJ19-P*_prpD2_*-Lba-P4N1-*gfp* | pXMJ19-P*_prpD2_*-Lba-Ec-*gfp* derivative with the P*_prpD2_* promoter variant P4 combined with the NCS variant N1 | This study |
| pXMJ19-P*_prpD2_*-Lba-P4N2-*gfp* | pXMJ19-P*_prpD2_*-Lba-Ec-*gfp* derivative with the P*_prpD2_* promoter variant P4 combined with the NCS variant N2 | This study |
| pXMJ19-P*_prpD2_*-Lba-P6N1-*gfp* | pXMJ19-P*_prpD2_*-Lba-Ec-*gfp* derivative with the P*_prpD2_* promoter variant P6 combined with the NCS variant N1 | This study |
| pXMJ19-P*_prpD2_*-Lba-P6N2-*gfp* | pXMJ19-P*_prpD2_*-Lba-Ec-*gfp* derivative with the P*_prpD2_* promoter variant P6 combined with the NCS variant N2 | This study |
| pXMJ19-P*_prpD2_*-mHb-P1N3-*gfp* | pXMJ19-P*_prpD2_*-mHb-Cg-*gfp* derivative with the P*_prpD2_* promoter variant P1 combined with the NCS variant N3 | This study |
| pXMJ19-P*_prpD2_*-mHb-P1N4-*gfp* | pXMJ19-P*_prpD2_*-mHb-Cg-*gfp* with the P*_prpD2_* promoter variant P1 combined with the NCS variant N4 | This study |
| pXMJ19-P*_prpD2_*-mHb-P2N3-*gfp* | pXMJ19-P*_prpD2_*-mHb-Cg-*gfp* derivative with the P*_prpD2_* promoter variant P2 combined with the NCS variant N3 | This study |
| pXMJ19-P*_prpD2_*-mHb-P2N4-*gfp* | pXMJ19-P*_prpD2_*-mHb-Cg-*gfp* derivative with the P*_prpD2_* promoter variant P2 combined with the NCS variant N4 | This study |
| pXMJ19-P*_prpD2_*-mHb-P4N3-*gfp* | pXMJ19-P*_prpD2_*-mHb-Cg-*gfp* derivative with the P*_prpD2_* promoter variant P4 combined with the NCS variant N3 | This study |
| pXMJ19-P*_prpD2_*-mHb-P4N4-*gfp* | pXMJ19-P*_prpD2_*-mHb-Cg-*gfp* derivative with the P*_prpD2_* promoter variant P4 combined with the NCS variant N4 | This study |
| pXMJ19-P*_prpD2_*-mHb-P6N3-*gfp* | pXMJ19-P*_prpD2_*-mHb-Cg-*gfp* derivative with the P*_prpD2_* promoter variant P6 combined with the NCS variant N3 | This study |
| pXMJ19-P*_prpD2_*-mHb-P6N4-*gfp* | pXMJ19-P*_prpD2_*-mHb-Cg-*gfp* derivative with the P*_prpD2_* promoter variant P6 combined with the NCS variant N4 | This study |
| pXMJ19-P*_prpD2_*-Lba-P6N1-*gfp*-*copA*1 | pXMJ19-P*_prpD2_*-Lba-P6N1-*gfp* derivative with mutation in *copA* for increasing plasmid copy number to 107 | This study |
| pXMJ19-P*_prpD2_*-Lba-P6N1-*gfp*-*copA*2 | pXMJ19-P*_prpD2_*-Lba-P6N1-*gfp* derivative with mutation in *copA* for increasing plasmid copy number to 107 | This study |
| pXMJ19-P*_prpD2_*-Lba-P6N1-*gfp*-*copA*3 | pXMJ19-P*_prpD2_*-Lba-P6N1-*gfp* derivative with mutation in *copA* for increasing plasmid copy number to 250 | This study |
| pXMJ19-P*_prpD2_*-Lba-P6N1-*gfp*-*copA*4 | pXMJ19-P*_prpD2_*-Lba-P6N1-*gfp* derivative with mutation in *copA* for increasing plasmid copy number to 318 | This study |
| pXMJ19-P*_prpD2_*-Lba-Ec | pXMJ19-P*_prpD2_*-Lba-Ec-*gfp* derivative with *gfp* removed | This study |
| pXMJ19-P*_prpD2_*-Lba-N1 | pXMJ19-P*_prpD2_*-Lba-Ec derivative with the optimized NCS variant (N1) | This study |
| pXMJ19-P*_prpD2_*-Lba-P6 | pXMJ19-P*_prpD2_*-Lba-Ec derivative with the optimized P*_prpD2_* promoter variant (P6) | This study |
| pXMJ19-P*_prpD2_*-Lba-P6N1 | pXMJ19-P*_prpD2_*-Lba-P6N1-*gfp* derivative with *gfp* removed | This study |
| pXMJ19-P*_prpD2_*-Lba-P6N1-*copA*1 | pXMJ19-P*_prpD2_*-Lba-P6N1-*gfp*-*copA*1 derivative with *gfp* removed | This study |

**Table S6.** Primers used for plasmid and library construction in this study.

| **Primer** | **Sequence (5'-3')** | **Plasmid** | **PCR template** | **Plasmid construction process** |
| --- | --- | --- | --- | --- |
| pXMJ19-F | AAGCTTGCATGCCTGCAG | pXMJ19-Lba-Ec-gfp | pXMJ19-Lba-Ec | Ligation of two PCR products via recombination |
| Lba-Ec-R | CGCTTTTTTAATCGCGGCCG |  |  |  |
| Lba-Ec-F1 | GCGATTAAAAAAGCGGGCGGTGGTGGTTCCGGC |  | pXMJ19-linker-gfp |  |
| gfp-R1 | CAGGCATGCAAGCTTTTATTTGTATAGTTCATCCATGCCA |  |  |  |
| pXMJ19-F | AAGCTTGCATGCCTGCAG | pXMJ19- Lba-Cg-gfp | pXMJ19-Lba-Cg | Ligation of two PCR products via recombination |
| Lba-Cg-R | TGCCTTCTTGATTGCTGCTGC |  |  |  |
| Lba-Cg-F1 | GCAATCAAGAAGGCAGGCGGTGGTGGTTCCGGC |  | pXMJ19-linker-gfp |  |
| gfp-R1 | CAGGCATGCAAGCTTTTATTTGTATAGTTCATCCATGCCA |  |  |  |
| pXMJ19-F | AAGCTTGCATGCCTGCAG | pXMJ19-mHb-Ec-gfp | pXMJ19-mHb-Ec | Ligation of two PCR products via recombination |
| mHb-Ec-R | CGCATCCGGTTTCATTTCGC |  |  |  |
| mHb-Ec-F1 | ATGAAACCGGATGCGGGCGGTGGTGGTTCCGGC |  | pXMJ19-linker-gfp |  |
| gfp-R1 | CAGGCATGCAAGCTTTTATTTGTATAGTTCATCCATGCCA |  |  |  |
| pXMJ19-F | AAGCTTGCATGCCTGCAG | pXMJ19-mHb-Cg-gfp | pXMJ19-mHb-Cg | Ligation of two PCR products via recombination |
| mHb-Cg-R | TGCATCAGGCTTCATTTCGCG |  |  |  |
| mHb-Cg-F1 | ATGAAGCCTGATGCAGGCGGTGGTGGTTCCGGC |  | pXMJ19-linker-gfp |  |
| gfp-R1 | CAGGCATGCAAGCTTTTATTTGTATAGTTCATCCATGCCA |  |  |  |
| pXMJ19-F | AAGCTTGCATGCCTGCAG | pXMJ19-rHb-Ec-gfp | pXMJ19-rHb-Ec | Ligation of two PCR products via recombination |
| rHb-Ec-R | CGCGGCCGCCGGTTTCAT |  |  |  |
| rHb-Ec-F1 | AAACCGGCGGCCGCGGGCGGTGGTGGTTCCGGC |  | pXMJ19-linker-gfp |  |
| gfp-R1 | CAGGCATGCAAGCTTTTATTTGTATAGTTCATCCATGCCA |  |  |  |
| pXMJ19-F | AAGCTTGCATGCCTGCAG | pXMJ19-rHb-Cg-gfp | pXMJ19-rHb-Cg | Ligation of two PCR products via recombination |
| rHb-Cg-R | TGCGGCTGCTGGCTTCATG |  |  |  |
| rHb-Cg-F1 | AAGCCAGCAGCCGCAGGCGGTGGTGGTTCCGGC |  | pXMJ19-linker-gfp |  |
| gfp-R1 | CAGGCATGCAAGCTTTTATTTGTATAGTTCATCCATGCCA |  |  |  |
| pXMJ19-F | AAGCTTGCATGCCTGCAG | pXMJ19-ScHb-Ec-gfp | pXMJ19-ScHb-Ec | Ligation of two PCR products via recombination |
| ScHb-Ec-R | TTTTTCCACGGTAATAAAAATAT |  |  |  |
| ScHb-Ec-F1 | ATTACCGTGGAAAAAGGCGGTGGTGGTTCCGGC |  | pXMJ19-linker-gfp |  |
| gfp-R1 | CAGGCATGCAAGCTTTTATTTGTATAGTTCATCCATGCCA |  |  |  |
| pXMJ19-F | AAGCTTGCATGCCTGCAG | pXMJ19-ScHb-Cg-gfp | pXMJ19-ScHb-Cg | Ligation of two PCR products via recombination |
| ScHb-Cg-R | CTTTTCCACGGTGATGAAGATAT |  |  |  |
| ScHb-Cg-F1 | ATCACCGTGGAAAAGGGCGGTGGTGGTTCCGGC |  | pXMJ19-linker-gfp |  |
| gfp-R1 | CAGGCATGCAAGCTTTTATTTGTATAGTTCATCCATGCCA |  |  |  |
| Lba-F | AGCTTAAAGGAGTTGAGAATGGTT | pXMJ19-Lba-gfp | The native Lba gene synthesized by Genscript | Ligation of two PCR products via recombination |
| Lba-R | GAACCACCACCGCCCTGCAGTGCCTTCTTAATAGCTGCTGC |  |  |  |
| YUAN-F | CTGCAGGGCGGTGGTG |  | pXMJ19-rHb-Cg-gfp |  |
| YUAN-R | TCTCAACTCCTTTAAGCTTAATTAA |  |  |  |
| mHb-F | TAAGCTTAAAGGAGTTGAGAATGGCACT | pXMJ19-mHb-gfp | The native mHb gene synthesized by Genscript | Ligation of two PCR products via recombination |
| mHb-R | GAACCACCACCGCCCTGCAGGGCATCGGGCTTCATCTC |  |  |  |
| YUAN-F | CTGCAGGGCGGTGGTG |  | pXMJ19-rHb-Cg-gfp |  |
| YUAN-R | TCTCAACTCCTTTAAGCTTAATTAA |  |  |  |
| rHb-F | TAAGCTTAAAGGAGTTGAGAATGGC | pXMJ19-rHb-gfp | The native rHb gene synthesized by Genscript | Ligation of two PCR products via recombination |
| rHb-R | GAACCACCACCGCCCTGCAGCGCAGCAGCCGGCTTCATCC |  |  |  |
| YUAN-F | CTGCAGGGCGGTGGTG |  | pXMJ19-rHb-Cg-gfp |  |
| YUAN-R | TCTCAACTCCTTTAAGCTTAATTAA |  |  |  |
| ScHb-F | AGCTTAAAGGAGTTGAGAATGCTAG | pXMJ19-ScHb-gfp | The native ScHb gene synthesized by Genscript | Ligation of two PCR products via recombination |
| ScHb-R | GAACCACCACCGCCCTGCAGTTTTTCAACAGTGATGAAGATGTCA |  |  |  |
| YUAN-F | CTGCAGGGCGGTGGTG |  | pXMJ19-rHb-Cg-gfp |  |
| YUAN-R | TCTCAACTCCTTTAAGCTTAATTAA |  |  |  |
| PprpD2-F | GGGCAACAGCTGATTGCCCTCTCCAGCGTCCAAGAATATGC | pXMJ19-P*_prpD2_*-Lba-Ec-gfp | Genomic DNA of *C. glutamicum* | Ligation of two PCR products via recombination |
| Lba-PprpD2-R | TTTTCGGTAAACGCCACCATTCTCAACTCCTTTGCACACCACACTAATTCTTTAAAAA |  |  |  |
| Lba-F1 | ATGGTGGCGTTTACCGAAAAACA |  | pXMJ19-Lba-Ec-gfp |  |
| P-R1 | AGGGCAATCAGCTGTTGCC |  |  |  |
| PprpD2-F | GGGCAACAGCTGATTGCCCTCTCCAGCGTCCAAGAATATGC | pXMJ19-P*_prpD2_*-mHb-Cg-gfp | Genomic DNA of *C. glutamicum* | Ligation of two PCR products via recombination |
| mHb-PprpD2-R | TCTGCTTCTGCCAGTGCCATTCTCAACTCCTTTAACGAACTAATGTTGCACACCACACTAATTCTTTAAAAA |  |  |  |
| mHb-F1 | ATGGCACTGGCAGAAGCA |  | pXMJ19-mHb-Cg-gfp |  |
| P-R1 | AGGGCAATCAGCTGTTGCC |  |  |  |
| Pgntk-F | GGGCAACAGCTGATTGCCCTGTATCAATGGAATCCGGGA | pXMJ19-P*_gntK_*-Lba-Ec-gfp | Genomic DNA of *C. glutamicum* | Ligation of two PCR products via recombination |
| Lba-Pgntk-R | TTTTCGGTAAACGCCACCATATGATATCTCCTTCTTAAAGTTCAG |  |  |  |
| Lba-F1 | ATGGTGGCGTTTACCGAAAAACA |  | pXMJ19-Lba-Ec-gfp |  |
| P-R1 | AGGGCAATCAGCTGTTGCC |  |  |  |
| Pgntk-F | GGGCAACAGCTGATTGCCCTGTATCAATGGAATCCGGGA | pXMJ19-P*_gntK_*-mHb-Cg-gfp | Genomic DNA of *C. glutamicum* | Ligation of two PCR products via recombination |
| M- Pgntk-R | TCTGCTTCTGCCAGTGCCATATGATATCTCCTTCTTAAAGTTCAG |  |  |  |
| mHb-F1 | ATGGCACTGGCAGAAGCAGA |  | pXMJ19-mHb-Cg-gfp |  |
| P-R1 | AGGGCAATCAGCTGTTGCC |  |  |  |
| Lba-P1-N1 | AACTTTTACAGTGACCTACATTGTCGTGCCGGCTGCTAGACTGGTGTGCAAAGGAGTTGAGAATGGTTGCATTTACAGAAAAACAAGACGCCCTAGTTAGCAGTAGCTTTGAAGCGTTTAA | pXMJ19-P*_prpD2_*-Lba-P1N1-gfp | pXMJ19-P*_prpD2_*-Lba-Ec-gfp | Ligation of two PCR products via recombination |
| NP-R1 | TCCGTATGGCAATGAAAGAC |  |  |  |
| NP-F2 | GTCTTTCATTGCCATACGGA |  |  |  |
| NP-R2 | AGGGCAATCAGCTGTTGCC |  |  |  |
| Lba-P1-N2 | AACTTTTACAGTGACCTACATTGTCGTGCCGGCTGCTAGACTGGTGTGCAAAGGAGTTGAGAATGGTGGCTTTCACTGAGAAACAGGACGCGCTCGTCAGCAGTAGCTTTGAAGCGTTTAA | pXMJ19-P*_prpD2_*-Lba-P1N2-gfp | pXMJ19-P*_prpD2_*-Lba-Ec-gfp | Ligation of two PCR products via recombination |
| NP-R1 | TCCGTATGGCAATGAAAGAC |  |  |  |
| NP-F2 | GTCTTTCATTGCCATACGGA |  |  |  |
| NP-R2 | AGGGCAATCAGCTGTTGCC |  |  |  |
| Lba-P3-N1 | AACTTTTACAGTGACCTACAATATGCGGGGATAGTGTAGAATGGTGTGCAAAGGAGTTGAGAATGGTTGCATTTACAGAAAAACAAGACGCCCTAGTTAGCAGTAGCTTTGAAGCGTTTAA | pXMJ19-P*_prpD2_*-Lba-P3N1-gfp | pXMJ19-P*_prpD2_*-Lba-Ec-gfp | Ligation of two PCR products via recombination |
| NP-R1 | TCCGTATGGCAATGAAAGAC |  |  |  |
| NP-F2 | GTCTTTCATTGCCATACGGA |  |  |  |
| NP-R2 | AGGGCAATCAGCTGTTGCC |  |  |  |
| Lba-P3-N2 | AACTTTTACAGTGACCTACAATATGCGGGGATAGTGTAGAATGGTGTGCAAAGGAGTTGAGAATGGTGGCTTTCACTGAGAAACAGGACGCGCTCGTCAGCAGTAGCTTTGAAGCGTTTAA | pXMJ19-P*_prpD2_*-Lba-P3N2-gfp | pXMJ19-P*_prpD2_*-Lba-Ec-gfp | Ligation of two PCR products via recombination |
| NP-R1 | TCCGTATGGCAATGAAAGAC |  |  |  |
| NP-F2 | GTCTTTCATTGCCATACGGA |  |  |  |
| NP-R2 | AGGGCAATCAGCTGTTGCC |  |  |  |
| Lba-P4-N1 | AACTTTTACAGTGACCTACATTAGGATTATCTAGGTTAAGATGGTGTGCAAAGGAGTTGAGAATGGTTGCATTTACAGAAAAACAAGACGCCCTAGTTAGCAGTAGCTTTGAAGCGTTTAA | pXMJ19-P*_prpD2_*-Lba-P4N1-gfp | pXMJ19-P*_prpD2_*-Lba-Ec-gfp | Ligation of two PCR products via recombination |
| NP-R1 | TCCGTATGGCAATGAAAGAC |  |  |  |
| NP-F2 | GTCTTTCATTGCCATACGGA |  |  |  |
| NP-R2 | AGGGCAATCAGCTGTTGCC |  |  |  |
| Lba-P4-N2 | AACTTTTACAGTGACCTACATTAGGATTATCTAGGTTAAGATGGTGTGCAAAGGAGTTGAGAATGGTGGCTTTCACTGAGAAACAGGACGCGCTCGTCAGCAGTAGCTTTGAAGCGTTTAA | pXMJ19-P*_prpD2_*-Lba-P4N2-gfp | pXMJ19-P*_prpD2_*-Lba-Ec-gfp | Ligation of two PCR products via recombination |
| NP-R1 | TCCGTATGGCAATGAAAGAC |  |  |  |
| NP-F2 | GTCTTTCATTGCCATACGGA |  |  |  |
| NP-R2 | AGGGCAATCAGCTGTTGCC |  |  |  |
| Lba-P6-N1 | AACTTTTACAGTGACCTACAGTCTTTCTTGTGTTATAATTTGGTGTGCAAAGGAGTTGAGAATGGTTGCATTTACAGAAAAACAAGACGCCCTAGTTAGCAGTAGCTTTGAAGCGTTTAA | pXMJ19-P*_prpD2_*-Lba-P6N1-gfp | pXMJ19-P*_prpD2_*-Lba-Ec-gfp | Ligation of two PCR products via recombination |
| NP-R1 | TCCGTATGGCAATGAAAGAC |  |  |  |
| NP-F2 | GTCTTTCATTGCCATACGGA |  |  |  |
| NP-R2 | AGGGCAATCAGCTGTTGCC |  |  |  |
| Lba-P6-N2 | AACTTTTACAGTGACCTACAGTCTTTCTTGTGTTATAATTTGGTGTGCAAAGGAGTTGAGAATGGTGGCTTTCACTGAGAAACAGGACGCGCTCGTCAGCAGTAGCTTTGAAGCGTTTAA | pXMJ19-P*_prpD2_*-Lba-P6N2-gfp | pXMJ19-P*_prpD2_*-Lba-Ec-gfp | Ligation of two PCR products via recombination |
| NP-R1 | TCCGTATGGCAATGAAAGAC |  |  |  |
| NP-F2 | GTCTTTCATTGCCATACGGA |  |  |  |
| NP-R2 | AGGGCAATCAGCTGTTGCC |  |  |  |
| mHb-P1-N3 | AACTTTTACAGTGACCTACATAGCCGGTTGGTGTGCTATATTGGTGTGCAACATTAGTTCGTTAAAGGAGTTGAGAATGGCTCTTGCAGAAGCCGATGACGGCGCTGTGGTTTTCGGCGAAGAACAAGAAGCACT | pXMJ19-P*_prpD2_*-mHb-P1N3-gfp | pXMJ19-P*_prpD2_*-mHb-Cg-gfp | Ligation of two PCR products via recombination |
| NP-R1 | TCCGTATGGCAATGAAAGAC |  |  |  |
| NP-F2 | GTCTTTCATTGCCATACGGA |  |  |  |
| NP-R2 | AGGGCAATCAGCTGTTGCC |  |  |  |
| mHb-P1-N4 | AACTTTTACAGTGACCTACATAGCCGGTTGGTGTGCTATATTGGTGTGCAACATTAGTTCGTTAAAGGAGTTGAGAATGGCACTGGCTGAAGCTGATGATGGCGCCGTGGTTTTCGGCGAAGAACAAGAAGCACT | pXMJ19-P*_prpD2_*-mHb-P1N4-gfp | pXMJ19-P*_prpD2_*-mHb-Cg-gfp | Ligation of two PCR products via recombination |
| NP-R1 | TCCGTATGGCAATGAAAGAC |  |  |  |
| NP-F2 | GTCTTTCATTGCCATACGGA |  |  |  |
| NP-R2 | AGGGCAATCAGCTGTTGCC |  |  |  |
| mHb-P2-N3 | AACTTTTACAGTGACCTACACGTTTTTAGCCTATGTTAAGCTGGTGTGCAACATTAGTTCGTTAAAGGAGTTGAGAATGGCTCTTGCAGAAGCCGATGACGGCGCTGTGGTTTTCGGCGAAGAACAAGAAGCACT | pXMJ19-P*_prpD2_*-mHb-P2N3-gfp | pXMJ19-P*_prpD2_*-mHb-Cg-gfp | Ligation of two PCR products via recombination |
| NP-R1 | TCCGTATGGCAATGAAAGAC |  |  |  |
| NP-F2 | GTCTTTCATTGCCATACGGA |  |  |  |
| NP-R2 | AGGGCAATCAGCTGTTGCC |  |  |  |
| mHb-P2-N4 | AACTTTTACAGTGACCTACACGTTTTTAGCCTATGTTAAGCTGGTGTGCAACATTAGTTCGTTAAAGGAGTTGAGAATGGCACTGGCTGAAGCTGATGATGGCGCCGTGGTTTTCGGCGAAGAACAAGAAGCACT | pXMJ19-P*_prpD2_*-mHb-P2N4-gfp | pXMJ19-P*_prpD2_*-mHb-Cg-gfp | Ligation of two PCR products via recombination |
| NP-R1 | TCCGTATGGCAATGAAAGAC |  |  |  |
| NP-F2 | GTCTTTCATTGCCATACGGA |  |  |  |
| NP-R2 | AGGGCAATCAGCTGTTGCC |  |  |  |
| mHb-P4-N3 | AACTTTTACAGTGACCTACACATGGTGCGTATCGTGTAAGTTGGTGTGCAACATTAGTTCGTTAAAGGAGTTGAGAATGGCTCTTGCAGAAGCCGATGACGGCGCTGTGGTTTTCGGCGAAGAACAAGAAGCACT | pXMJ19-P*_prpD2_*-mHb-P4N3-gfp | pXMJ19-P*_prpD2_*-mHb-Cg-gfp | Ligation of two PCR products via recombination |
| NP-R1 | TCCGTATGGCAATGAAAGAC |  |  |  |
| NP-F2 | GTCTTTCATTGCCATACGGA |  |  |  |
| NP-R2 | AGGGCAATCAGCTGTTGCC |  |  |  |
| mHb-P4-N4 | AACTTTTACAGTGACCTACACATGGTGCGTATCGTGTAAGTTGGTGTGCAACATTAGTTCGTTAAAGGAGTTGAGAATGGCACTGGCTGAAGCTGATGATGGCGCCGTGGTTTTCGGCGAAGAACAAGAAGCACT | pXMJ19-P*_prpD2_*-mHb-P4N4-gfp | pXMJ19-P*_prpD2_*-mHb-Cg-gfp | Ligation of two PCR products via recombination |
| NP-R1 | TCCGTATGGCAATGAAAGAC |  |  |  |
| NP-F2 | GTCTTTCATTGCCATACGGA |  |  |  |
| NP-R2 | AGGGCAATCAGCTGTTGCC |  |  |  |
| mHb-P6-N3 | AACTTTTACAGTGACCTACATTGGTTCTTCTGCTGTAACGTGGTGTGCAACATTAGTTCGTTAAAGGAGTTGAGAATGGCTCTTGCAGAAGCCGATGACGGCGCTGTGGTTTTCGGCGAAGAACAAGAAGCACT | pXMJ19-P*_prpD2_*-mHb-P6N3-gfp | pXMJ19-P*_prpD2_*-mHb-Cg-gfp | Ligation of two PCR products via recombination |
| NP-R1 | TCCGTATGGCAATGAAAGAC |  |  |  |
| NP-F2 | GTCTTTCATTGCCATACGGA |  |  |  |
| NP-R2 | AGGGCAATCAGCTGTTGCC |  |  |  |
| mHb-P6-N4 | AACTTTTACAGTGACCTACATTGGTTCTTCTGCTGTAACGTGGTGTGCAACATTAGTTCGTTAAAGGAGTTGAGAATGGCACTGGCTGAAGCTGATGATGGCGCCGTGGTTTTCGGCGAAGAACAAGAAGCACT | pXMJ19-P*_prpD2_*-mHb-P6N4-gfp | pXMJ19-P*_prpD2_*-mHb-Cg-gfp | Ligation of two PCR products via recombination |
| NP-R1 | TCCGTATGGCAATGAAAGAC |  |  |  |
| NP-F2 | GTCTTTCATTGCCATACGGA |  |  |  |
| NP-R2 | AGGGCAATCAGCTGTTGCC |  |  |  |
| CTF1 | CAAACCCCCGTGCGAGCTACTAACTCATATGCACGGGGGCC | pXMJ19-P*_prpD2_*-Lba-P6N1-gfp-copA1 | pXMJ19-P*_prpD2_*-Lba-P6N1-gfp | Ligation of two PCR products via recombination |
| PLB-R1 | GCGTTATCCCCTGATTCTGTGG |  |  |  |
| PLB-F2 | CCACAGAATCAGGGGATAACGC |  |  |  |
| PLB-R2 | GTAGCTCGCACGGGGGTTT |  |  |  |
| CF1 | CAAACCCCCGTGCGAGCTACAACTCATATGCACGGGGGCC | pXMJ19-P*_prpD2_*-Lba-P6N1-gfp-copA2 | pXMJ19-P*_prpD2_*-Lba-P6N1-gfp | Ligation of two PCR products via recombination |
| PLB-R1 | GCGTTATCCCCTGATTCTGTGG |  |  |  |
| PLB-F2 | CCACAGAATCAGGGGATAACGC |  |  |  |
| PLB-R2 | GTAGCTCGCACGGGGGTTT |  |  |  |
| ACF1 | CAAACCCCCGTGCGAGCTAACAACTCATATGCACGGGGGCC | pXMJ19-P*_prpD2_*-Lba-P6N1-gfp-copA3 | pXMJ19-P*_prpD2_*-Lba-P6N1-gfp | Ligation of two PCR products via recombination |
| PLB-R1 | GCGTTATCCCCTGATTCTGTGG |  |  |  |
| PLB-F2 | CCACAGAATCAGGGGATAACGC |  |  |  |
| PLB-R2 | GTAGCTCGCACGGGGGTTT |  |  |  |
| CGF1 | CAAACCCCCGTGCGAGCTACGAACTCATATGCACGGGGGCC | pXMJ19-P*_prpD2_*-Lba-P6N1-gfp-copA4 | pXMJ19-P*_prpD2_*-Lba-P6N1-gfp | Ligation of two PCR products via recombination |
| PLB-R1 | GCGTTATCCCCTGATTCTGTGG |  |  |  |
| PLB-F2 | CCACAGAATCAGGGGATAACGC |  |  |  |
| PLB-R2 | GTAGCTCGCACGGGGGTTT |  |  |  |
| B-Lba-F1 | CGGCCGCGATTAAAAAAGCGTAAAAGCTTGCATGCCTGCAGG | pXMJ19-P*_prpD2_*-Lba-Ec | pXMJ19-P*_prpD2_*-Lba-Ec-gfp | Ligation of two PCR products via recombination |
| B-R1 | GGGAGGGTTACGTGTCCGATAC |  |  |  |
| B-F2 | GTATCGGACACGTAACCCTCCC |  |  |  |
| B-Lba-R2 | CGCTTTTTTAATCGCGGCC |  |  |  |
| B-Lba-F1 | CGGCCGCGATTAAAAAAGCGTAAAAGCTTGCATGCCTGCAGG | pXMJ19-P*_prpD2_*-Lba-N1 | pXMJ19-P*_prpD2_*-Lba-Ec-gfp-N1 | Ligation of two PCR products via recombination |
| B-R1 | GGGAGGGTTACGTGTCCGATAC |  |  |  |
| B-F2 | GTATCGGACACGTAACCCTCCC |  |  |  |
| B-Lba-R2 | CGCTTTTTTAATCGCGGCC |  |  |  |
| B-Lba-F1 | CGGCCGCGATTAAAAAAGCGTAAAAGCTTGCATGCCTGCAGG | pXMJ19-P*_prpD2_*-Lba-P6 | pXMJ19-P*_prpD2_*-Lba-Ec-gfp-P6 | Ligation of two PCR products via recombination |
| B-R1 | GGGAGGGTTACGTGTCCGATAC |  |  |  |
| B-F2 | GTATCGGACACGTAACCCTCCC |  |  |  |
| B-Lba-R2 | CGCTTTTTTAATCGCGGCC |  |  |  |
| B-Lba-F1 | CGGCCGCGATTAAAAAAGCGTAAAAGCTTGCATGCCTGCAGG | pXMJ19-P*_prpD2_*-Lba-P6N1 | pXMJ19-P*_prpD2_*-Lba-P6N1-gfp | Ligation of two PCR products via recombination |
| B-R1 | GGGAGGGTTACGTGTCCGATAC |  |  |  |
| B-F2 | GTATCGGACACGTAACCCTCCC |  |  |  |
| B-Lba-R2 | CGCTTTTTTAATCGCGGCC |  |  |  |
| B-Lba-F1 | CGGCCGCGATTAAAAAAGCGTAAAAGCTTGCATGCCTGCAGG | pXMJ19-P*_prpD2_*-Lba-P6N1-copA1 | pXMJ19-P*_prpD2_*-Lba-P6N1-gfp-copA1 | Ligation of two PCR products via recombination |
| B-R1 | GGGAGGGTTACGTGTCCGATAC |  |  |  |
| B-F2 | GTATCGGACACGTAACCCTCCC |  |  |  |
| B-Lba-R2 | CGCTTTTTTAATCGCGGCC |  |  |  |
| Lba-NF1 | GCAAAGGAGTTGAGAATGGTNGCNTTYACNGARAARCARGAYGCNCTNGTNAGCAGTAGCTTTGAAGCGTTTAA | NCS library of Lba-Ec | pXMJ19-P*_prpD2_*-Lba-Ec-gfp | Ligation of two PCR products via recombination |
| Lba-NF2 | GCAAAGGAGTTGAGAATGGTNGCNTTYACNGARAARCARGAYGCNTTRGTNAGCAGTAGCTTTGAAGCGTTTAA |  |  |  |
| NR1 | GCATTTATCAGGGTTATTGTCTCA |  |  |  |
| NF2 | TGAGACAATAACCCTGATAAATGC |  |  |  |
| NR2 | ACCATTCTCAACTCCTTTGCA |  |  |  |
| mHb-NF1 | TTAAAGGAGTTGAGAATGGCNCTNGCNGARGCNGAYGAYGGNGCNGTNGTNTTCGGCGAAGAACAAGAAGC | NCS library of mHb-Cg | pXMJ19-P*_prpD2_*-mHb-Cg-gfp | Ligation of two PCR products via recombination |
| mHb-NF2 | TTAAAGGAGTTGAGAATGGCNTTRGCNGARGCNGAYGAYGGNGCNGTNGTNTTCGGCGAAGAACAAGAAGC |  |  |  |
| NR1 | GCATTTATCAGGGTTATTGTCTCA |  |  |  |
| NF2 | TGAGACAATAACCCTGATAAATGC |  |  |  |
| NR3 | GCCATTCTCAACTCCTTTAACGA |  |  |  |
| PprpD2-Lba-F1 | AACTTTTACAGTGACCTACANNNNNNNNNNNNNNNNTANNNTGGTGTGCAAAGGAGTTGAGAAT | P*_prpD2_* promoter library of Lba-Ec | pXMJ19-P*_prpD2_*-Lba-Ec-gfp | Ligation of two PCR products via recombination |
| PprpD2-R | GTCTTGTAGTTCCCGTCATCTTTG |  |  |  |
| PprpD2-pXMJ19-F | CAAAGATGACGGGAACTACAA |  |  |  |
| PprpD2-pXMJ19-R | TGTAGGTCACTGTAAAAGTTGTGTA |  |  |  |
| PprpD2-mHb-F1 | AACTTTTACAGTGACCTACANNNNNNNNNNNNNNNNTANNNTAACATTAGTTCGTTAAAGGAGTTGA | P*_prpD2_* promoter library of mHb-Cg | pXMJ19-P*_prpD2_*-mHb-Cg-gfp | Ligation of two PCR products via recombination |
| PprpD2-R | GTCTTGTAGTTCCCGTCATCTTTG |  |  |  |
| PprpD2-pXMJ19-F | CAAAGATGACGGGAACTACAA |  |  |  |
| PprpD2-pXMJ19-R | TGTAGGTCACTGTAAAAGTTGTGTA |  |  |  |
| gntk-up-F | AGCTCGGTACCCGGGGATCCCCTTCTCCCCTCGTGTGCTC | pK18∆gntK | Genomic DNA of *C. glutamicum* | Ligation of three PCR products via recombination |
| gntk-up-R | GTCTTATCCTTTCTTTGGTGGCG |  |  |  |
| gntk-down-F | CACCAAAGAAAGGATAAGACTGGCTAGTCCAGGTTGGCA |  |  |  |
| gntk-down-R | GCCTGCAGGTCGACTCTAGACTTCCCCTGCGGTGACTG |  |  |  |
| Pk18-F1 | TCTAGAGTCGACCTGCAGGC |  | pK18*mobsacB* |  |
| Pk18-R1 | GGATCCCCGGGTACCGAGCT |  |  |  |
| PprpD2-up-F | AATCTCGTGATGGCAGGTTG ATCTTGTCCATCCTGTTCGC | pCas9gRNA-∆prpDBC2 | Genomic DNA of *C. glutamicum* | Ligation of five PCR products via recombination |
| PprpD2-up-R | TTCCGCAGAACGGTGGGT |  |  |  |
| PprpD2-down-F | GCACCCACCGTTCTGCGGAAGTACAACGGCGAGGAGCAG |  |  |  |
| PprpD2-down-R | CAGTTATTGGTGCCCTTCGAGTTTCCATTCTCACCACCAA |  |  |  |
| P-F1 | TCGAAGGGCACCAATAACTGC |  | pCas9gRNA-ccdB |  |
| P-R1 | CTTTTACTTTCACCAGCGTTTCTG |  |  |  |
| P-F2 | AACGCTGGTGAAAGTAAAAGATGC |  |  |  |
| P-R2 | TCAAATCCGGCTTCCTGGATTGAATTACACTGTACCTGTTGCGTC |  |  |  |
| P-F3 | ATCCAGGAAGCCGGATTTGAGTTTTAGAGCTAGAAATAGCAAG |  |  |  |
| P-R3 | CAACCTGCCATCACGAGATTTTC |  |  |  |
| Pk18-gntk-F | TGGCTGAGCAGTTCAACTTGGA | PCR verification of the 504 bp deletion of *gntK* | | |
| Pk18-gntk-R | GGGGAAAACCCTGGCGTTACCC |  |  |  |
| VBCDF | CTTTGGCATGGGATCTCATTGTCAC | PCR verification of the 3,530 bp deletion of *prpDBC2* | | |
| VBCDR | CTGGAGTACCTGCAGCAGACGC |  |  |  |

**Supplementary references**

1. Jakoby M, Ngouoto-Nkili C-E, Burkovski A. Construction and application of new *Corynebacterium glutamicum* vectors. Biotechnol Tech. 1999;13:437-441.
2. Schäfer A, Tauch A, Jäger W, Kalinowski J, Thierbach G, Pühler A. Small mobilizable multi-purpose cloning vectors derived from the *Escherichia coli* plasmids pK18 and pK19: selection of defined deletions in the chromosome of *Corynebacterium glutamicum*. Gene. 1994;145:69-73.
3. Liu J, Liu M, Shi T, Sun G, Gao N, Zhao X, Guo X, Ni X, Yuan Q, Feng J, Liu Z, Guo Y, Chen J, Wang Y, Zheng P, Sun J. CRISPR-assisted rational flux-tuning and arrayed CRISPRi screening of an L-proline exporter for L-proline hyperproduction. Nat Commun. 2022;13:891.
